# VRK1 co-delivery mitigates DNA clustering by BAF in TFAMoplex transfection

**DOI:** 10.1016/j.mtbio.2025.102588

**Published:** 2025-11-21

**Authors:** Christina Greitens, Philip Maurer, Selen Balkan, Jean-Christophe Leroux, Michael Burger

**Affiliations:** Institute of Pharmaceutical Sciences, Department of Chemistry and Applied Biosciences, ETH Zurich, Vladimir-Prelog-Weg 3, 8093, Zurich, Switzerland

**Keywords:** Non-viral gene delivery, BAF phosphorylation, VRK1, Protein-based transfection, Intracellular imaging, Nanoparticles

## Abstract

In non-viral gene delivery, DNA entering the cytoplasm is rapidly bound by barrier-to-autointegration factor (BAF) and surrounded by nuclear envelope-like membranes. This process is thought to mitigate the translocation of the DNA from the cytoplasm through the nuclear pores limiting the transfection of interphase cells. To prevent BAF clustering, we developed the protein-based gene delivery system TFAMoplex that uses the human mitochondrial transcription factor A (TFAM) which forms nanoparticles with DNA. In previous studies, we covalently fused the endogenous BAF inactivating enzyme vaccinia-related kinase 1 (VRK1) to TFAM, which, however, did not decrease BAF-mediated clustering of the transfected DNA. Here, we describe a modified TFAMoplex where VRK1 is linked to TFAM via a SpyTag/SpyCatcher linker system (SpyTFAMoplex). With this approach, quantitative image analysis, performed with transfected enhanced green fluorescent protein (EGFP)-BAF overexpressing HeLa cells, showed that cytoplasmic BAF clustering can be counteracted. A SpyTFAMoplex version comprising a kinase-inactive VRK1 mutant (dSpyTFAMoplex) exhibited twice the number of clusters per cell. The decrease in BAF clusters achieved with the active VRK1 system was accompanied by lower transfection efficiency in HeLa cells with significantly less (−72 %) mean fluorescence intensity of reporter gene expression compared to dSpyTFAMoplex. These data suggest that VRK1 in the SpyTFAMoplex interferes with BAF's DNA-binding ability, potentially impairing the mitotic nuclear entry pathway of exogenous DNA. While our study highlights the potential of co-delivering enzymes with DNA, further vector engineering is required to directly guide cytosolic DNA into the nucleus of interphase cells.

## Highlights

• New version of TFAM-VRK1 conjugation in TFAMoplex decreases DNA clustering by BAF.
• Reporter gene expression with VRK1 is lower than with dead VRK1.
• It is hypothesized that VRK1 impairs the mitotic nuclear entry pathway.

## Graphical abstract

**Figure:**
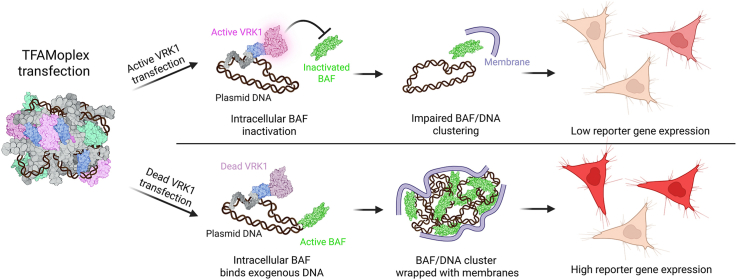

## 1 Introduction

In non-viral DNA delivery, strategies to control the cytoplasmic fate of the DNA and to promote active gene transfer into the nucleus remain insufficiently investigated. In contrast to RNA therapeutics (*e.g.*, messenger RNA or short interfering RNA), where cytoplasmic delivery of the cargo is sufficient [1], DNA requires nuclear uptake to enable gene transcription. The DNA cargo size is usually larger than 250 bp hindering passive diffusion through the crowded cytosol [[2], [3], [4]] and through the nuclear pore complex (NPC), which has a pore size of around 40 nm [[5], [6], [7]]. Cytoplasmic mobility of large nucleic acids can be enhanced by binding of motor proteins promoting transport along microtubules [[8], [9], [10]]. Transcription factors may bind to specific sequences on the DNA and promote with their nuclear localization signals active transport through the NPC [[11], [12], [13], [14]]. Nucleic acids also enter the nucleus during mitotic nuclear breakdown, accounting for the transfection efficiencies achieved in dividing *vs*. non-dividing cells [15,16]. Haraguchi et al. [17] showed that exogenous DNA can associate with chromatin in telophase and is incorporated in the reforming nucleus, followed by transgene expression in the subsequent interphase. As many cells in the human body are non-dividing or slowly dividing, efficient gene delivery through the intact nucleus is essential for gene therapy applications.

Cells have evolved strategies to retain cytosolic DNA from entering the nucleus [18]. One major obstacle is the barrier-to-autointegration factor (BAF), a 10-kDa, mostly homodimeric protein that is present both, in the nucleus, particularly localized at the inner nuclear leaflet, and in the cytoplasm [19]. It binds double-stranded DNA (dsDNA) intra- and intermolecularly, independent of the DNA sequence [20]. BAF is crucial for nuclear envelope reassembly in telophase by interacting with both, the chromosomal DNA and LAP2, emerin and MAN1 (LEM)-domain proteins [[21], [22], [23], [24], [25], [26]].

However, BAF also binds exogenous DNA immediately after its cytosolic appearance [21,27,28]. The DNA molecules can be clumped together by BAF, accompanied by the recruitment of nuclear envelope-like membranes to form a dense nucleoprotein structure, termed cytoplasmic BAF cluster. While this clustering may protect cytoplasmic DNA from degradation [[29], [30], [31], [32], [33]] and prolong the residence time of the DNA inside the cell, it may partially prevent DNA uptake through the NPC and present a major obstacle to the transfection of non-dividing cells [34].

BAF's binding to DNA is modulated by phosphorylation via the human vaccinia-related kinase 1 (VRK1). This has been, for example, characterized by Nichols et al. [35] and Burger et al. [34], and particularly occurs at the onset of mitosis [22]. The dephosphorylation of BAF by protein-phosphatase 2A is thought to be regulated by LEM4 at mitotic exit [36,37]. DNA viruses can (i) avoid the contact of their genome with BAF in the cytoplasm (*e.g.* Parvoviridae) [38], (ii) possess enzymes to inactivate it (*e.g.* Vaccinia virus) [[39], [40], [41], [42]], or (iii) use BAF to their advantage as transduction promoter (*e.g.* Retroviruses) [27,43]. In contrast, non-viral vectors generally lack mechanisms to counteract BAF. However, protein-based vectors offer the possibility to incorporate functional proteins [44], such as the BAF inactivating VRK1, to protect the DNA inside the cell.

**Fig. 1:**
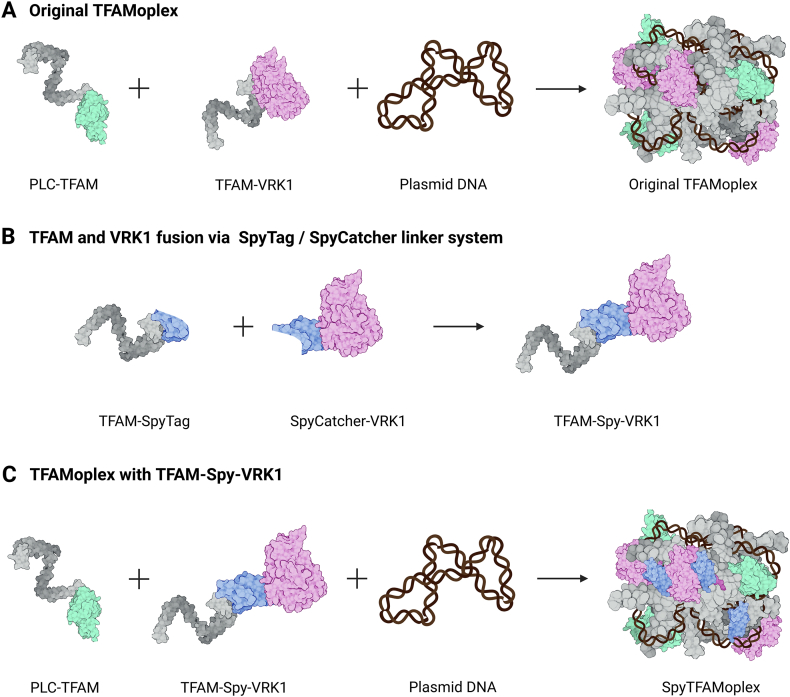
Composition of the TFAMoplex versions. **A**) Original TFAMoplex with TFAM (grey), PLC (green), VRK1 (pink) **B**) SpyTag/SpyCatcher linker system (blue) **C**) SpyTFAMoplex with PLC-TFAM and TFAM-Spy-VRK1. Adapted and modified under terms of the CC-BY 4.0 license, Honrath et al. 2025 [47], published by Elsevier.

We recently reported the protein-based transfection agent TFAMoplex that can efficiently transfect cells, even at low amounts of DNA, short incubation times, and in full serum [10,[45], [46], [47]]. It is built on the human protein mitochondrial transcription factor A (TFAM) (Fig. 1A) that binds DNA in a sequence-unspecific manner and forms nanoparticles of about 100 nm in diameter with plasmid DNA (pDNA) [48]. For successful transfection, TFAM or modified versions of TFAM are genetically fused to two functional proteins: *Listeria monocytogenes* phospholipase C (PLC) [[49], [50], [51]] (PLC-TFAM) which destabilizes the endosomal membrane to escape the endo-lysosomal pathway [46] and VRK1 (TFAM-VRK1) which was added to inhibit BAF [35,52]. However, our previous work showed that the TFAMoplex comprising a kinase-dead version of VRK1 (dVRK1, D177A) transfected HeLa cells equally well as the active VRK1 construct [45]. Moreover, the VRK1 construct could not prevent BAF cluster formation with the transfected DNA [53].

In this study, we assess whether cytoplasmic DNA can be protected from BAF-mediated clustering by co-delivering different forms of VRK1 fusion proteins in the TFAMoplex, and how this influences transfection efficiency. First, we examine whether TFAM-VRK1 remains associated with the DNA inside the cell. Second, we seek to enhance the cytoplasmic activity of VRK1 by producing TFAM and VRK1 separately and subsequently coupling the proteins via the SpyTag/SpyCatcher system [54,55]. This split protein can be joined together through a spontaneous covalent isopeptide bond formation that irreversibly links two fusion proteins. This introduces a linker between TFAM and VRK1 potentially enhancing VRK1's activity inside the TFAMoplex (Fig. 1B). Our aim is to shed light on how co-delivered human enzymes can influence the transfection process, particularly the characteristics of exogenous DNA clustering by BAF and the role of cytoplasmic DNA retention.

## 2 Results and discussion

### 2.1 Original TFAMoplex initially colocalizes with EGFP-BAF clusters

**Fig. 2:**
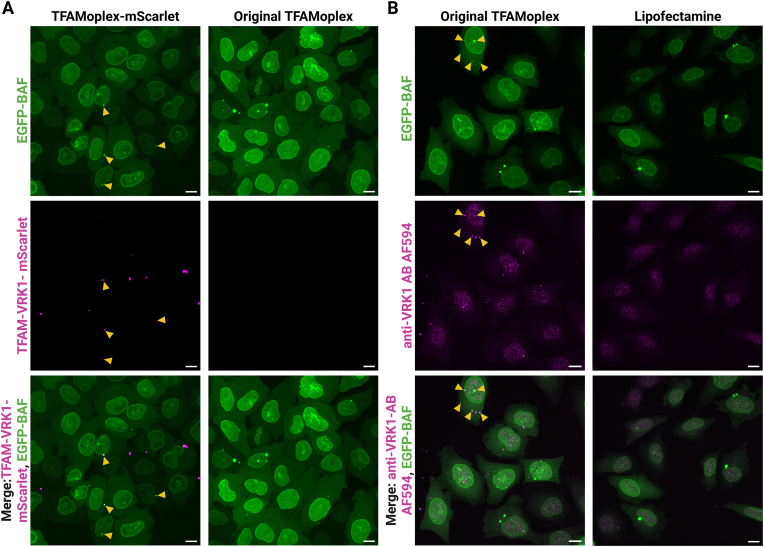
Colocalization of the original TFAMoplex with EGFP-BAF clusters 4 h after transfection. Confocal microscopy images shown as z-projections of maximum intensity of 35 stacks with 0.3 μm slice thickness. TFAMoplexes prepared with PLC-TFAM, TFAM-VRK1-mScarlet or TFAM-VRK1 (original TFAMoplex) and enhanced blue fluorescent protein (EBFP) encoding pDNA. EGFP-BAF cells were transfected for 4 h in 100 % fetal bovine serum (FBS) with 200 ng EBFP-pDNA/mL. Excitation at 20 % laser intensity for 200 ms in all channels. **A**) TFAMoplex-mScarlet or original TFAMoplex transfected cells were washed, fixed and imaged. Magenta: TFAM-VRK1-mScarlet (intensity: 300–1000), green: EGFP-BAF (intensity: 50–1000). **B**) Original TFAMoplex or Lipofectamine transfected cells were washed, fixed, permeabilized, and immunostained with a primary AB mouse anti-human VRK1 and a secondary AB goat anti-mouse AF594. Magenta: anti-VRK1 AB AF594 (intensity: 100–700), green: EGFP-BAF (intensity: 100–700). Orange arrowheads indicate colocalization with bright EGFP-BAF foci. Scale bars: 10 μm.

**Fig. 3:**
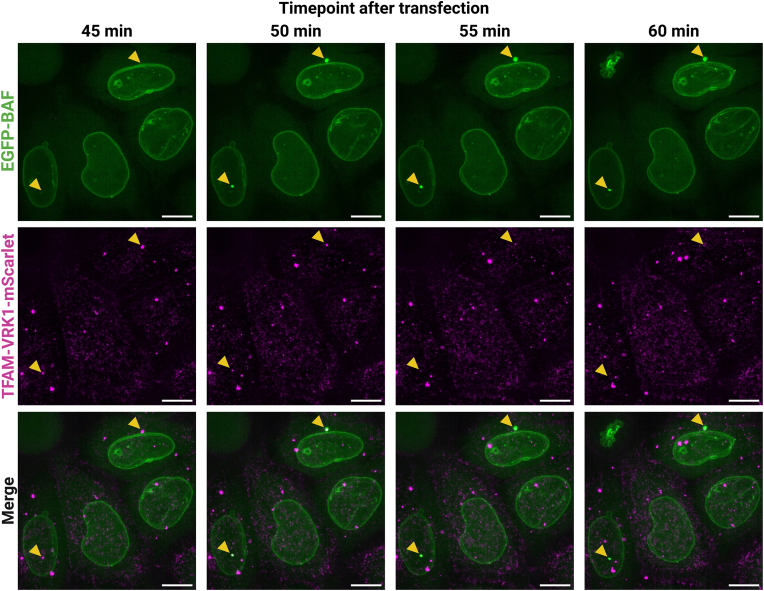
Timelapse imaging of TFAMoplex-mScarlet transfected EGFP-BAF cells. Zoom-ins of confocal microscopy images shown as z-projections of maximum intensity of 18 stacks with 0.5 μm slice thickness. TFAMoplex-mScarlet was prepared with PLC-TFAM, TFAM-VRK1-mScarlet and EBFP-pDNA. EGFP-BAF cells were transfected in imaging medium with 400 ng EBFP-pDNA/mL. Excitation at 5 % laser intensity for 100 ms in both channels. Recording started 30 min after transfection acquiring z-stacks every 5 min. Magenta: TFAM-VRK1-mScarlet (intensity: 120–450), green: EGFP-BAF (intensity: 100–300). Orange arrowheads indicate colocalization of TFAM-VRK1-mScarlet signal with EGFP-BAF clusters. Scale bars: 10 μm. The video is provided in Video S1.

Our previous study [53] showed that TFAMoplex transfection triggered the formation of intracellular BAF clusters containing the delivered labeled DNA. A possible explanation for the inability of VRK1 to protect the DNA from BAF clustering is its rapid dissociation from the DNA inside the cytoplasm. Consequently, we herein assessed whether the co-delivered VRK1 remained associated with the DNA during the transfection process. Therefore, TFAM-VRK1 was labeled with the fluorescent protein mScarlet (TFAM-VRK1-mScarlet) and used to transfect HeLa cells stably overexpressing EGFP-BAF (EGFP-BAF cells) [26]. Fig. 2A shows the colocalization of the TFAMoplex-mScarlet signal with the bright EGFP-BAF clusters. Colocalization was more pronounced 4 h than 24 h after transfection (Fig. S1) indicating that the delivered proteins of the TFAMoplex might degrade or dissociate from the DNA over time. Such mechanisms are further supported by timelapse imaging, showing the appearance of EGFP-BAF clusters that were initially enriched with TFAM-VRK1-mScarlet, but largely lost the mScarlet signal within 1 h (Fig. 3, Video S1, Table S1).

One explanation for the disappearance of the TFAM-VRK1-mScarlet signal in EGFP-BAF clusters could be a competition for DNA binding sites between BAF and TFAM. Both have DNA binding affinities in the nanomolar range [48,56] but BAF is more abundant inside the cell and might displace the TFAM from the DNA, when BAF is not phosphorylated.

To confirm the presence of VRK1 in BAF clusters with an additional visualization method, we transfected EGFP-BAF cells with unlabeled original TFAMoplexes or Lipofectamine and immunostained the cells 4 h after transfection with an anti-VRK1 antibody (AB) and a secondary AB coupled to Alexa Fluor (AF)594 (Fig. 2B). Note that this AB can detect both endogenous and exogenous VRK1 moieties. As expected, anti-VRK1 signal from endogenous and potentially exogenous VRK1 in the nuclear region was detected in both TFAMoplex and Lipofectamine transfection conditions. While no colocalization of EGFP-BAF clusters with anti-VRK1 signal was observed in the Lipofectamine condition, few EGFP-BAF clusters colocalized with anti-VRK1 staining following TFAMoplex transfection. However, most EGFP-BAF clusters showed weak or no immunostaining, possibly due to the absence of TFAM-VRK1 or the dense and membrane-wrapped architecture of the BAF clusters [28], which might hinder the accessibility of the ABs to the target sites.

Taken together, the presence of VRK1 in BAF clusters, particularly at early timepoints, indicates that the TFAM proteins are, at least partially, associated with the DNA inside the cytoplasm. The data also suggest that VRK1, at this stage, does not hinder BAF from binding and clustering of DNA. However, in gel mobility shift assays (Fig. S2 and reference [45]), TFAM-VRK1 protects DNA from BAF clustering. In the absence of VRK1, DNA was clumped and retained by BAF in the gel wells. The addition of VRK1 permitted DNA to migrate in the gel similarly as in the absence of BAF. The discrepancy between the *in cellulo* and the gel mobility results suggests that the activity of the delivered VRK1 in the TFAMoplex is likely impaired in the cell.

In summary, we conclude that TFAM-VRK1 remains at least partially complexed to DNA during BAF cluster formation and dissociates subsequently. However, the kinase of the original TFAMoplexes was unable to protect the DNA from BAF clustering, potentially due to restricted motion or steric hindrance. Consequently, we sought to improve VRK1 activity by incorporating a linker domain between TFAM and VRK1 with the aim of enhancing the activity of VRK1 in the TFAMoplex. For this purpose, we used the SpyTag and SpyCatcher linker system [55] and produced the TFAM-SpyTag and SpyCatcher-(d)VRK1 constructs separately (Fig. 1) from *E. coli*. The SDS-PAGE (Fig. S3A) confirmed that a 15 min incubation of TFAM-SpyTag with SpyCatcher-VRK1 or SpyCatcher-dVRK1 led to covalent bond formation. To assess the activity of the TFAM-Spy-VRK1 or TFAM-Spy-dVRK1 constructs *in vitro*, we performed a gel mobility shift assay by incubating the proteins with pDNA and BAF (Fig. S4). In the presence of BAF, the DNA was retained in the wells in the TFAM-Spy-dVRK1 condition, while with TFAM-Spy-VRK1, the DNA migrated in the gel. The particles formed with pDNA and TFAM-Spy-VRK1 or TFAM-Spy-dVRK1 had a hydrodynamic diameter of ca. 100 nm (Fig. S5), comparable to the original TFAMoplex [45]. Moreover, neither the SpyTFAMoplex nor the dSpyTFAMoplex showed cytotoxicity at concentrations corresponding to the usual transfection conditions (Fig. S6). In the subsequent sections, we use the term “SpyTFAMoplex” for the system containing PLC-TFAM, TFAM-SpyTag and SpyCatcher-VRK1 (Fig. 1C) and the term “dSpyTFAMoplex” for the complexes prepared with PLC-TFAM, TFAM-SpyTag and SpyCatcher-dVRK1.

### 2.2 SpyTFAMoplex reduces BAF clustering

**Fig. 4:**
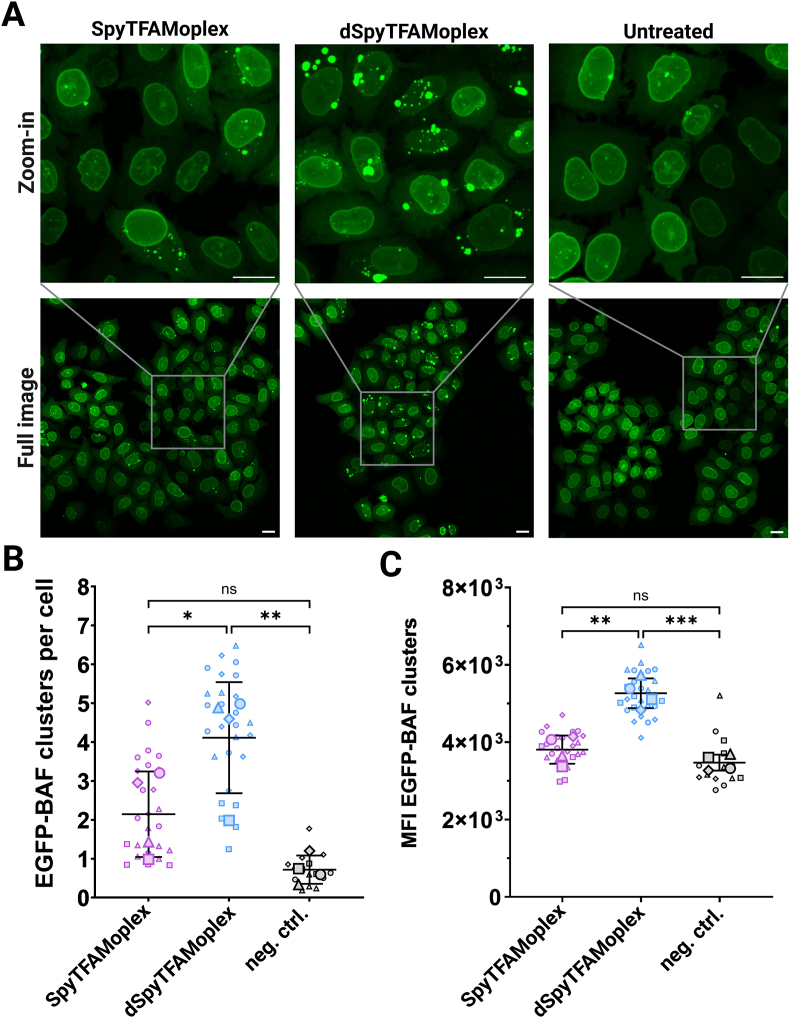
EGFP-BAF cells one day after transfection. Cells were incubated overnight with SpyTFAMoplex or dSpyTFAMoplex with 400 ng mScarlet-pDNA/mL medium and imaged after one day. Particle formation was performed with 1 μM TFAM-SpyTag and 0.8 μM SpyCatcher-VRK1 or 0.8 μM SpyCatcher-dVRK1, and 1 μM PLC-TFAM. **A**) Confocal microscopy images displayed as z-projections of maximum intensity of 31 slices with 0.5 μm slice thickness. Excitation at 20 % laser intensity for 200 ms. Green: EGFP-BAF (intensity:100–2700). Scale bars: 20 μm. **B**) EGFP-BAF clusters per cell. **C**) MFI EGFP-BAF clusters. Each small symbol in the superplots represents one image. 3–9 images per condition in one biological experiment. Large symbols represent the means of all images in one biological experiment. Total four independent biological experiments were performed. Mean ± SD (N = 4). Statistical significance was analyzed with Repeated Measures one-way ANOVA with Tukey's multiple comparison test. ∗p < 0.05, ∗∗p < 0.01, ∗∗∗p < 0.001. SpyTFAMoplex: 2895 cells, 6006 EGFP-BAF clusters; dSpyTFAMoplex: 3001 cells, 12,531 EGFP-BAF clusters; neg. ctrl. (non-transfected cells): 1932 cells, 1271 EGFP-BAF clusters.

To evaluate the intracellular interaction of BAF with DNA during SpyTFAMoplex or dSpyTFAMoplex transfection, we transfected EGFP-BAF cells with 400 ng mScarlet-pDNA/mL overnight and imaged them one day after transfection with confocal microscopy (Fig. 4). EGFP-BAF cells transfected with SpyTFAMoplex showed less and smaller EGFP-BAF clusters with remarkably lower EGFP intensity compared to dSpyTFAMoplex (Fig. 4A). Via automated image analysis, we quantified the number of clusters per cell by segmentation of bright EGFP-BAF foci and measured the EGFP intensity (Fig. 4B and C). With dSpyTFAMoplex 4.1 ± 1.4 clusters per cell were detected, that is approx. twice the number of clusters per cell observed with SpyTFAMoplex (2.1 ± 1.1). The clusters were also significantly brighter with dSpyTFAMoplex compared to SpyTFAMoplex (mean fluorescence intensity (MFI) 5269 ± 285 *vs.* 3812 ± 365, respectively) indicating that the few clusters observed in the presence of VRK1 contained less EGFP-BAF. EGFP-BAF clusters per cell and MFI in the SpyTFAMoplex condition did not differ significantly from the non-transfected control.

The data suggest that the VRK1 delivered with the SpyTFAMoplex reduced BAF-dependent DNA clustering. To understand the DNA/VRK1/BAF interaction in more detail, we transfected EGFP-BAF cells with Cy3-DNA and imaged the cells at various timepoints.

### 2.3 EGFP-BAF clusters disappear over time due to VRK1

**Fig. 5:**
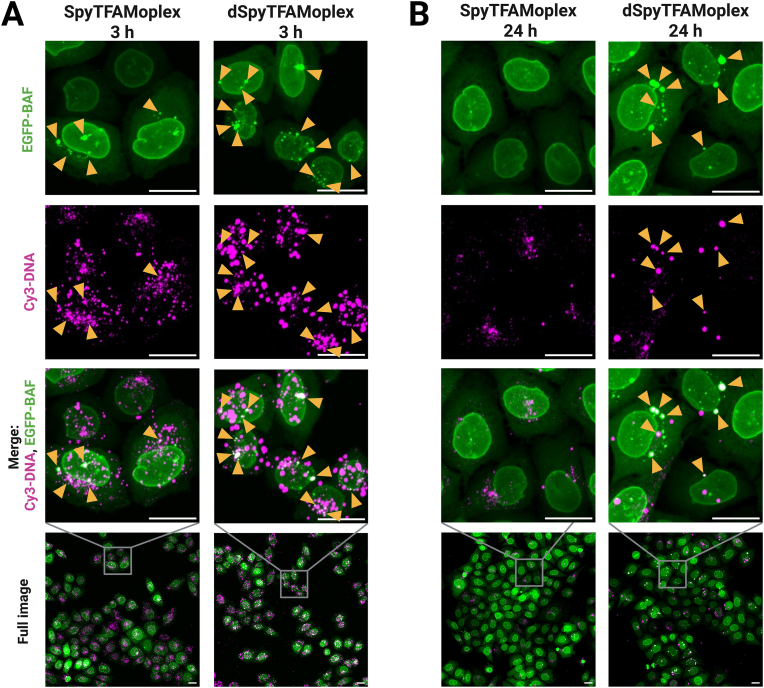
EGFP-BAF cells transfected with Cy3-DNA fixed after 3 h (**A**) and 24 h (**B**). Confocal microscopy zoom-ins and full images displayed as z-projections of maximum intensity of 31 slices with 0.5 μm slice thickness. EGFP-BAF cells were incubated for 30 min with SpyTFAMoplex or dSpyTFAMoplex with 400 ng Cy3-DNA/mL medium. Cells were washed and further incubated until fixation after a total of 3 or 24 h post transfection. Particles were prepared with PLC-TFAM, TFAM-SpyTag and SpyCatcher-VRK1 or SpyCatcher-dVRK1 and Cy3-DNA. Magenta: Cy3-DNA (intensity: 150–600), green: EGFP-BAF (intensity: 150–2000). Excitation at 20 % laser intensity for 200 ms in all channels. Orange arrowheads indicate colocalization of Cy3-DNA with bright EGFP-BAF foci. Scale bars: 20 μm.

To assess the fate of exogenous DNA within BAF clusters, we incubated EGFP-BAF cells with 400 ng Cy3-DNA/mL medium complexed with SpyTFAMoplex or dSpyTFAMoplex for 30 min and fixed the cells either 3 or 24 h post transfection (Fig. 5). We first assessed the total Cy3-DNA signal per cell, irrespective of whether the signal was located on the cell surface or internalized. The overall Cy3 signal was comparable between both transfection conditions, indicating that the two systems associate with the cells to a similar extent. Notably, the Cy3 signal was more pronounced at 3 h than at 24 h. For the SpyTFAMoplex condition, the Cy3 foci at 24 h appeared fainter and smaller compared to those observed with dSpyTFAMoplex. The SpyTFAMoplex induced considerable EGFP-BAF cluster formation 3 h after transfection. However, these clusters were barely detected after 24 h, suggesting their disassembly over time. In contrast, dSpyTFAMoplex samples showed strong clustering at both timepoints.

We also assessed EGFP-BAF clustering 3 h (Fig. S7) and 24 h (Fig. S8) post transfection at lower DNA loads, *i.e.* 200, 40, and 4 ng/mL. As expected, Cy3-DNA cell association as well as EGFP-BAF clustering after 3 h were less pronounced when less DNA was applied. In the SpyTFAMoplex conditions, almost no EGFP-BAF clusters were observed at the 24 h timepoint independent of the applied DNA load whereas with dSpyTFAMoplex, EGFP-BAF clustering was comparable at both timepoints within the respective DNA load.

**Fig. 6:**
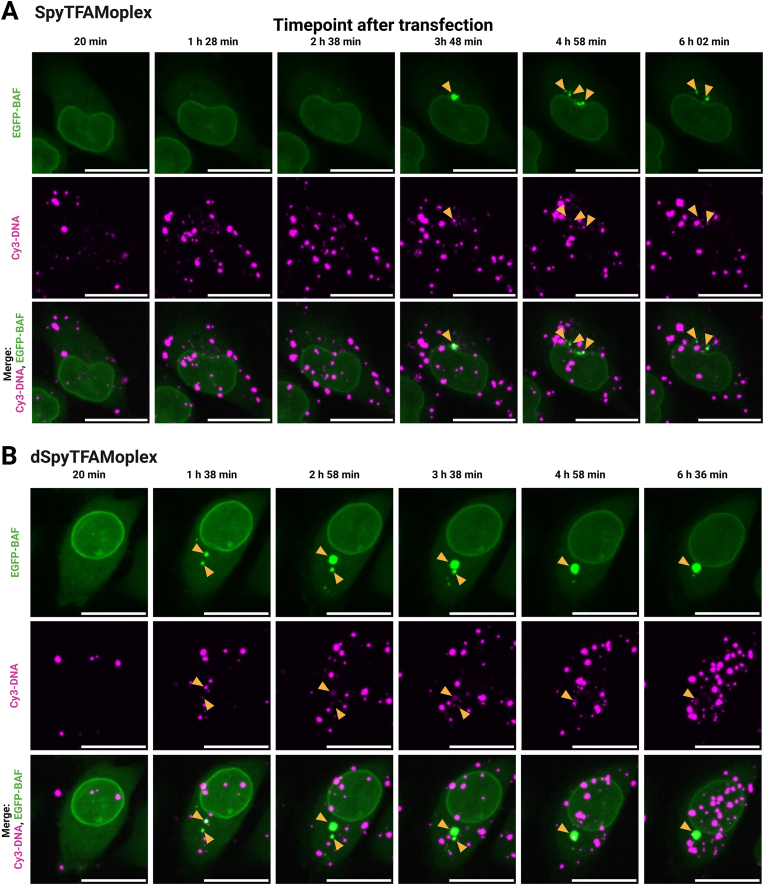
Timelapse imaging of EGFP-BAF cells transfected with SpyTFAMoplex (**A**) and dSpyTFAMoplex (**B**) at 200 ng Cy3-DNA/mL imaging medium. Confocal microscopy zoom-ins shown as z-projections of maximum intensity of 17 stacks with 0.5 μm slice thickness. Excitation at 5 % laser intensity for 100 ms in all channels. Live cell imaging started 20 min after transfection acquiring z-stacks every 2 min. Magenta: Cy3-DNA (intensity: 100–700), green: EGFP-BAF (intensity: 100–700). Scale bars: 20 μm. Orange arrowheads indicate formation and fate of bright EGFP-BAF foci. Videos are provided in Videos S2 and S3.

To evaluate the disappearance of the BAF clusters from the early to the late timepoints, we performed live cell imaging of EGFP-BAF cells transfected with 400 ng Cy3-DNA/mL acquiring z-stacks every 2 min (Fig. 6, Video S2 and S3). With the SpyTFAMoplex, the clustering was less pronounced than with dSpyTFAMoplex and the few EGFP-BAF foci containing Cy3-DNA often dissolved into smaller foci until almost complete dissociation (Fig. 6A, Video S2). Cluster formation for dSpyTFAMoplex, however, was extensive and comparable to the original TFAMoplex shown in Fig. 3 and Video S1. The intense EGFP-BAF clusters mostly persisted over the entire acquired timeframe and small EGFP-BAF foci often seemed to fuse with larger foci (Fig. 6B, Video S3). The timelapse data suggest that VRK1 in the SpyTFAMoplex actively inhibits BAF in the cytoplasm after initial clustering occurred, resulting in the dissociation of BAF from the DNA or the dissolution of the clusters. The fact that EGFP-BAF recruitment to exogenous DNA is still observed at early timepoints could be explained by a reversible inactivation of the kinase in the endolysosomal condition. Moreover, other cellular factors could be involved in cluster formation and dissolution, resulting in complex kinetics, and further work is needed to clarify the mechanism.

We analyzed the effect of the active VRK1 on the HeLa cell phosphorylation status after 4 h incubation with 400 ng pDNA/mL medium with the SpyTFAMoplex and the dSpyTFAMoplex (Fig. S9, Table S3). Fig. S9A shows that phosphorylation was generally more pronounced with SpyTFAMoplex. However, hyperphosphorylated phosphopeptides did not relate to VRK1 substrates [57,58] and BAF was not significantly more phosphorylated with SpyTFAMoplex *vs*. dSpyTFAMoplex (Table S3). From these data, one can hypothesize that this assay is not sensitive enough to detect local changes in BAF phosphorylation as the delivered VRK1 may only affect BAF phosphorylation in the vicinity of the transfected DNA [42,59,60].

### 2.4 Characterization of transfected intracellular DNA

**Fig. 7:**
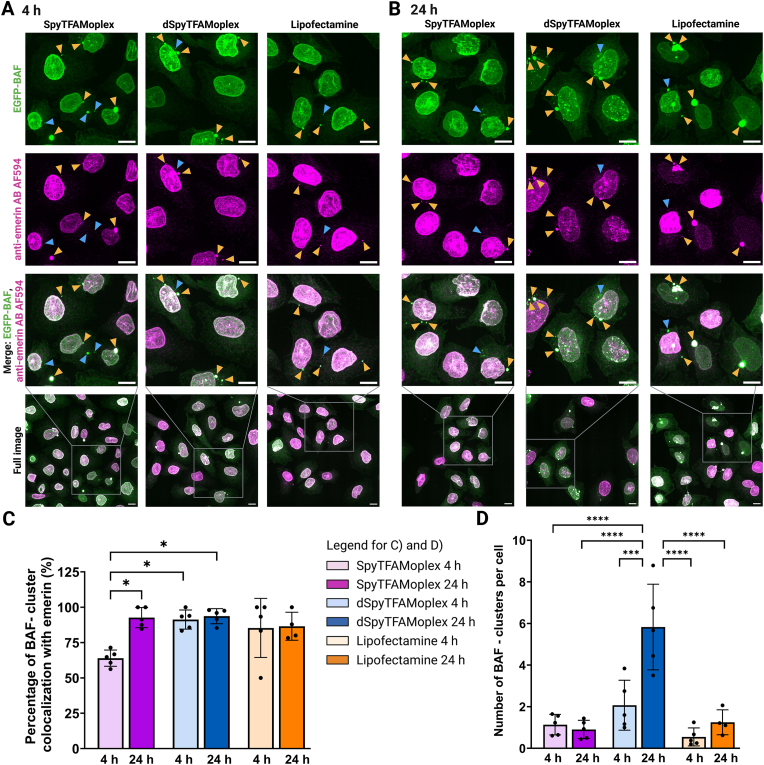
Emerin immunostaining of transfected EGFP-BAF cells. Confocal microscopy zoom-ins and full images displayed as z-projections of maximum intensity of 38 slices of 0.2 μm slice thickness. Cells were incubated for 30 min with SpyTFAMoplex, dSpyTFAMoplex or Lipofectamine at 400 ng EBFP-pDNA/mL medium. Cells were washed and further incubated until fixation after 4 h (**A**) and 24 h (**B**). Immunostaining was performed using primary AB mouse anti-emerin and a secondary AB goat anti-mouse AF594. Magenta: anti-emerin AB AF594 (intensity: 100–500), green: EGFP-BAF (intensity:115–500). Scale bars: 10 μm. Orange arrowheads indicate colocalization of EGFP-BAF foci with anti-emerin AB AF594 signal. Blue arrowheads indicate emerin-negative EGFP-BAF foci **C**) Percentage of EGFP-BAF clusters that colocalize with anti-emerin AB AF594 staining. Bright EGFP-BAF clusters with an anti-emerin AB AF594 MFI >160.81 were considered as emerin-positive. **D**) Number of BAF clusters per cell. **C**) and **D**) each data point represents one image. Mean ± SD of 4–5 images per condition in one biological experiment. Statistical significance was analyzed with Ordinary one-way ANOVA with Tukey's multiple comparison test. ∗p < 0.05, ∗∗∗p < 0.001, ∗∗∗∗p < 0.0001. SpyTFAMoplex 4 h: 117 cells, 123 EGFP-BAF clusters; SpyTFAMoplex 24 h: 73 cells, 66 EGFP-BAF clusters; dSpyTFAMoplex 4 h: 68 cells, 134 EGFP-BAF clusters; dSpyTFAMoplex 24 h: 65 cells, 375 EGFP-BAF clusters; Lipofectamine 4 h: 68 cells, 37 EGFP-BAF clusters; Lipofectamine 24 h: 70 cells, 87 EGFP-BAF clusters.

Generally, BAF-bound DNA is surrounded by nuclear envelope-like structures and are positive for LEM-domain proteins, especially the transmembrane protein emerin [[21], [22], [23], [24], [25], [26],^61^]. To determine whether the few EGFP-BAF clusters induced by SpyTFAMoplex transfection were also positive for a nuclear envelope marker, we performed anti-emerin immunostainings (anti-emerin AB AF594) in transfected EGFP-BAF cells at 4 and 24 h post transfection, alongside dSpyTFAMoplex and Lipofectamine controls (Fig. 7). As expected, we observed strong colocalization of EGFP-BAF with emerin at the nuclear rim. Most EGFP-BAF clusters were positive for emerin regardless of the transfection condition or time point of analysis (orange arrowheads in Fig. 7A and B). This suggests that emerin is recruited early after BAF accumulation to the transfected DNA, indicating the formation of cytoplasmic, membrane-wrapped nucleoprotein structures.

However, quantitative colocalization analysis (characterized in Fig. S10 and S11) revealed that with SpyTFAMoplex 4 h after transfection, only 64.0 % ± 5.8 % of bright EGFP-BAF foci colocalized with bright anti-emerin AB AF594 signal, which was significantly lower than SpyTFAMoplex at 24 h (92.7 % ± 7.1 %) and dSpyTFAMoplex at 4 h (91.3 % ± 6.7 %) and 24 h (93.7 % ± 6.7 %) (Fig. 7C). Also, the control Lipofectamine condition showed a trend toward higher colocalization percentages (85.3 % ± 20.9 at 4 h and 86.5 % ± 9.9 at 24 h). The reduced emerin recruitment to EGFP-BAF clusters in the SpyTFAMoplex condition 4 h after transfection suggests that VRK1 partially counteracts the membrane formation around BAF-bound DNA (blue arrowheads showing emerin-negative EGFP-BAF foci in Fig. 7A and B).

Fig. 7D displays the number of EGFP-BAF clusters per cell. The dSpyTFAMoplex condition at 24 h had the highest number of clusters per cell among all transfection conditions, consistent with Fig. 4B. While the SpyTFAMoplex showed almost a similar number of clusters per cell at 4 and 24 h timepoints (1.1 ± 0.5 and 0.9 ± 0.4, respectively), dSpyTFAMoplex revealed an increase in the number of clusters per cell from the 4 to the 24 h timepoint (2.1 ± 1.2 and 5.8 ± 2.1, respectively). This trend was also observed in the Lipofectamine group, though not significantly (0.5 ± 0.4 at 4 h and 1.3 ± 0.6 at 24 h). Note that upon combination of the data in Fig. 7C and D, the few EGFP-BAF clusters per cell in the SpyTFAMoplex condition that persisted until 24 h after transfection were mostly emerin-positive while the proportion of emerin-negative clusters decreased. Thus, the EGFP-BAF clusters at 24 h in the SpyTFAMoplex condition resemble the emerin characteristics of the other transfection conditions.

**Fig. 8:**
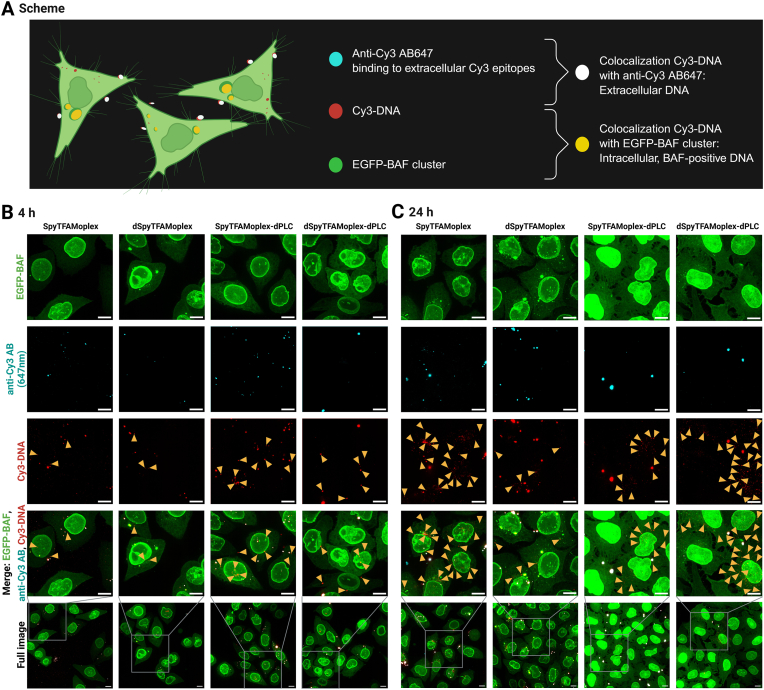
**A**) Scheme of the anti-Cy3 AB647 counterstaining assay with Cy3-DNA transfected EGFP-BAF cells. Confocal microscopy zoom-ins and full images of EGFP-BAF cells transfected with 400 ng Cy3-DNA/mL medium for 30 min, washed, further incubated and fixed after 4 h (**B**) or 24 h (**C**), and stained. Fluorescence channels displayed as z-projections of maximum intensity of 30–38 slices with 0.2 μm slice thickness. Cyan: anti-Cy3 AB647 (intensity:160–500), red: Cy3-DNA (intensity: 120–500), green: EGFP-BAF (intensity:120–500). Orange arrowheads indicate double-negative Cy3-DNA signals. Scale bars: 10 μm.

The reduced DNA clustering by BAF with SpyTFAMoplex observed above could result from diminished cell surface association of the complexes and/or diminished endosomal escape. To distinguish intracellular Cy3-DNA from Cy3-DNA signal originating from the cell membrane, we applied an anti-Cy3 AB counterstaining assay recently reported by our group [53]. To this end, EGFP-BAF cells were transfected with Cy3-DNA, and a cell impermeable anti-Cy3 AB was applied on fixed but not permeabilized cells after 4 and 24 h to only counterstain Cy3 epitopes on the cell surface. This approach allowed to distinguish three populations associated with the cells: extracellular DNA, EGFP-BAF-negative intracellular DNA, and EGFP-BAF-positive intracellular DNA. With the applied coloring, the colocalization of Cy3-DNA objects (red) with EGFP-BAF foci (green) appears yellow, while colocalization with anti-Cy3 AB (cyan) appears white (Fig. 8A). The latter events are localized on the cell surface and are thus always BAF-negative. Double-negative Cy3 signals correspond to DNA signals that were either retained in the endolysosomal system or evaded EGFP-BAF clustering in the cytoplasm or nucleus.

As a control, the staining was first performed with EGFP-BAF cells after transfection with Lipofectamine (Fig. S12). Next, cells were transfected with SpyTFAMoplex, dSpyTFAMoplex, and SpyTFAMoplexes containing a catalytically inactive mutant of PLC (dPLC) (Fig. 8). In the dPLC construct, the tryptophan at the active site (residue 52) is replaced with glycine (W52G) [45]. Consequently, SpyTFAMoplex-dPLC and dSpyTFAMoplex-dPLC particles are unable to escape the endolysosomal system, preventing the formation of EGFP-BAF clusters.

As expected, both SpyTFAMoplex-dPLC and dSpyTFAMoplex-dPLC never showed EGFP-BAF-positive Cy3 signals, neither 4 h nor 24 h after transfection, indicating that negligible endosomal escape has occurred. In contrast, EGFP-BAF-positive Cy3 signals were observed after 4 and 24 h in the Lipofectamine (Fig. S12A and B) and dSpyTFAMoplex transfection conditions and to a minor extent in the SpyTFAMoplex conditions (Fig. 8B and C), consistent with the observations in Fig. 5.

Double-negative Cy3 signals (*i.e.* intracellular Cy3-DNA not clustered by EGFP-BAF) at the 4 h timepoint were comparable among all transfection conditions, *i.e.* with active or dVRK1, with active or dPLC, or Lipofectamine (orange arrowheads in Fig. 8B, Fig. S12A) and likely corresponded to Cy3-DNA trapped in endolysosomes.

By 24 h however, the double-negative Cy3 signals in the dPLC conditions appeared smaller and fainter (orange arrowheads in Fig. 8C) than after 4 h, suggesting progressive DNA clearance in endolysosomes. Note that in such cases, free Cy3 fluorophores dissociated from degraded DNA may still produce diffuse signals [62].

Similarly, many faint and small double-negative Cy3 signals were observed in the SpyTFAMoplex condition with active PLC after 24 h (orange arrowheads in Fig. 8C). These types of signals were less pronounced in dSpyTFAMoplex 24 h condition, since most intracellular Cy3-DNA was clustered by EGFP-BAF.

We conclude that endosomal escape was PLC-dependent, which is in accordance with the findings of Klipp et al., 2025 [46]. In this work, an assay based on fluorescently labeled galectins that localize at ruptured endosomes was employed to assess the ability of various TFAMoplex versions to escape the endolysosomal compartment. The data suggested that an improved TFAMoplex system can enhance endosomal leakiness by releasing the PLC from the TFAMoplex in the endolysosomal compartment. The improved TFAMoplex was, in our previous study, not compared to a TFAMoplex with dPLC. Consequently, our results herein indicate that an active PLC is required to ensure cytoplasmic delivery. We hypothesize though, that the DNA molecules that were transfected with active VRK1 and active PLC (SpyTFAMoplex) escaped the endolysosomal system but were seemingly degraded when evading clustering by BAF.

In summary, the stepwise analysis of Cy3-DNA populations in EGFP-BAF cells at 4 and 24 h after transfection, including dPLC controls, suggests that DNA cell association, particle uptake, and likely also endosomal escape are comparable for SpyTFAMoplex and dSpyTFAMoplex. Thus, we hypothesize that the difference in the cytoplasmic response between the two is driven by the kinase activity in the SpyTFAMoplex.

The observation that BAF-negative pDNA in the cytoplasm may be more susceptible to clearance than BAF-positive DNA led to the hypothesis that non-BAF-clustered DNA could be translocated into lysosomes, e.g. through autophagocytosis [29]. Therefore, we analyzed colocalization of MFP488-labeled pDNA with Lysotracker DeepRed in transfected cells (Fig. S13). As expected, the small and faint labeled DNA signals in the SpyTFAMoplex condition after 24 h strongly colocalized with the Lysotracker staining. This localization within acidic cellular compartments suggests that a large proportion of cytoplasmic, BAF-negative DNA (Fig. 8C) undergoes degradation.

### 2.5 Reporter gene expression is decreased with SpyTFAMoplex transfection compared to dSpyTFAMoplex

**Fig. 9:**
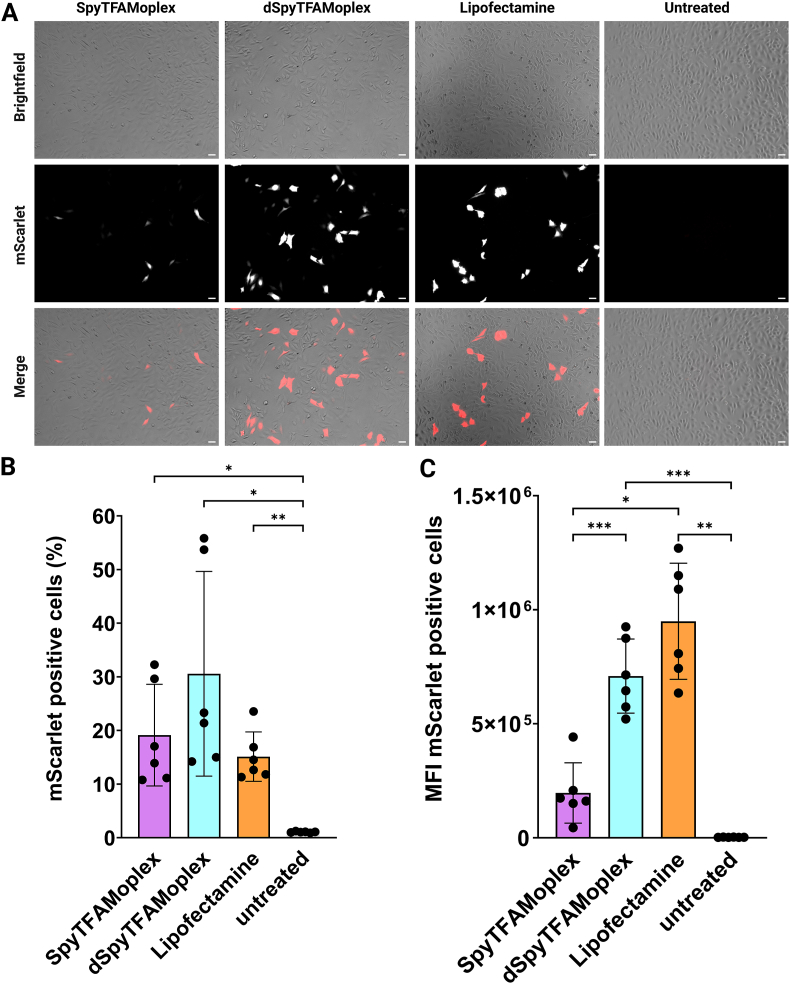
Transfection efficiency with SpyTFAMoplex, dSpyTFAMoplex, and Lipofectamine. HeLa cells were transfected with 200 ng mScarlet-pDNA/mL medium for 30 min and analyzed after 24 h. **A**) Representative Widefield microscope images showing mScarlet expression. Scale bars: 50 μm. **B**) Percentage of mScarlet expressing HeLa cells. **C)** MFI of mScarlet-positive cells. Significance was assessed with Repeated Measures one-way ANOVA. Mean ± SD (N = 6). ∗p < 0.05, ∗∗p < 0.01, ∗∗∗p < 0.001.

To assess transfection efficiency and reporter protein fluorescence intensity, HeLa cells were transfected with SpyTFAMoplex, dSpyTFAMoplex, and Lipofectamine (200 ng mScarlet-pDNA/mL medium). MScarlet expression was analyzed one day after transfection with widefield microscopy (Fig. 9A) and flow cytometry (Fig. 9B and C). The dSpyTFAMoplex condition yielded the highest transfection efficiency (30.6 % ± 19.1 %) compared to SpyTFAMoplex (19.1 % ± 9.5 %) and Lipofectamine (15.1 % ± 4.6 %), though not statistically significant. Interestingly, in terms of MFI of the mScarlet-positive cell populations, dSpyTFAMoplex outperformed the SpyTFAMoplex significantly (7.1 × 10^5^ ± 1.6 × 10^5^ *vs.* 2.0 × 10^5^ ± 1.3 × 10^5^, respectively), while Lipofectamine transfection showed the highest MFI (9.5 × 10^5^ ± 2.5 × 10^5^). The 3.5 times higher MFI of cells transfected with dSpyTFAMoplex *vs.* the SpyTFAMoplex indicates that more DNA reached the nucleus when VRK1 was inactive.

While the transfection data of the dSpyTFAMoplex in Fig. 9 are consistent with the results of the original TFAMoplex in our previous work [53], the almost absent DNA clustering by BAF after 24 h (Fig. 4) in the presence of active VRK1 together with the low reporter gene MFI (Fig. 9), indicates that VRK1 in the SpyTFAMoplex mitigates the transfection efficiency, stressing to further explore how the mechanisms used by viruses to transduce cells could be better adapted to synthetic systems. Our former work [47] indeed showed that adeno-associated virus (AAV) transduction outperformed the TFAMoplex transfection in terms of number of transfected cells but, interestingly, the TFAMoplex surpassed AAVs regarding transgene expression per cell. This suggests that in HeLa cells, AAVs mediate less efficient expression of internalized genes than TFAMoplexes, through a pathway that remains to be elucidated. One major nuclear uptake route contributing to successful non-viral transfection in dividing cells was assessed in detail by Haraguchi et al., 2022 [17] who showed that small fragments from disassembling BAF clusters can be incorporated into the nucleus during nuclear envelope reassembly in mitosis. Consequently, the absence of BAF clusters might reduce this pathway for nuclear DNA entry and lead to an overall lower transfection. Unfortunately, we currently cannot distinguish the extent of direct DNA uptake through the NPC from the mitotic pathways. It is therefore at present not possible to assess if the presence of active VRK1 during transfection is altering the efficiency of NPC-mediated nuclear uptake.

## 3 Conclusion

Non-viral DNA delivery is often hindered by low transfection efficiencies *in vivo*. In fact, most of the DNA reaching the cytoplasm becomes sequestered within BAF-dependent, membrane-bound structures [28]. These clusters can contain thousands of DNA molecules [63], which are largely prevented from accessing the nucleus through the NPC [34]. Partial disassembly of BAF clusters has been observed during mitosis, allowing a fraction of the DNA to enter the reforming nucleus [17]. However, the vast majority remains confined within the clusters in the cytoplasm [63], while DNA signals that clearly localize to the nuclear lumen remain barely detectable [62]. Most cells in the body are non-dividing, and even mitotically active cells divide at a much slower rate than immortalized cancer cell lines. Therefore, preventing BAF-dependent retention of DNA in the cytoplasm represents a key step in developing more effective non-viral DNA delivery vectors.

This study suggests for the first time that DNA clustering by BAF can be counteracted by introducing active human VRK1 in a protein-based transfection system. We show that the co-delivery of proteins effectively influences how specific intracellular machineries interact with the exogenous DNA. Transfection of EGFP-BAF overexpressing HeLa cells with the SpyTFAMoplex containing PLC-TFAM, TFAM-SpyTag and SpyCatcher-VRK1 resulted in a significantly fainter and lower number of EGFP-BAF foci than with the inactive kinase variant (Fig. 4) or the benchmark transfection agent Lipofectamine 3000.

The imaging data of transfected EGFP-BAF cells suggest that VRK1 mitigates the persistent formation of nuclear envelope-like nucleoprotein structures, potentially by phosphorylating BAF, although this could not be directly evidenced in this work.

Strikingly, the incorporation of active VRK1 in the SpyTFAMoplex showed a detrimental effect on reporter gene expression intensity (Fig. 9). Regarding the mitigated clustering, we conclude that BAF plays a pivotal role in protecting exogenous DNA from degradation and enabling nuclear entry during nuclear envelope breakdown in mitosis. This mitotic nuclear entry may strongly contribute to the successful transfection of synthetic transfection systems, i.e. lipoplexes or polyplexes, but may only work in dividing cells. The potential positive effect of BAF inhibition on successful transfection in interphase, *i.e.* through the NPC, remains to be elucidated.

Several factors may influence effective interphase transfection in the presence of VRK1. First, we observed that some DNA molecules are still bound by BAF at endosomal escape before clusters begin to dissolve (Fig. 5, Fig. 6**,** Video S2 and S3). This early clustering might reduce DNA mobility in the cytoplasm in a crucial moment for the transfection outcome. Second, the size and disassembly of the SpyTFAMoplex inside the cytoplasm remains to be investigated. The nuclear envelope poses a barrier that is difficult to overcome [[64], [65], [66]] and the 100 nm-sized SpyTFAMoplex is too large to pass the nuclear pore. Inefficient SpyTFAMoplex disassembly might thus hinder nuclear uptake of the DNA, even in the absence of BAF clustering. Potentially, TFAM could be engineered to dissociate from the DNA inside the cytoplasm to allow NPC-mediated DNA translocation. Lastly, cytoplasmic DNA can be degraded, as previously discussed in section 2.4 (Fig. S13) and DNA clearance might be enhanced in the absence of BAF-dependent clustering [29].

While physical non-viral gene delivery methods may not encounter such obstacles [67,68], interphase transfection should be improved in protein-based vector design and future developments should focus on a range of strategies. First, attenuating cytoplasmic DNA clearance is crucial and was recently reviewed by Zhang et al. [69]. Strategies include nucleic acid modifications, the effective encapsulation of DNA rendering it inaccessible for degrading enzymes or the inhibition of specific nucleases. Second, regulating DNA complex disassembly inside the cell, and third, facilitating NPC-mediated uptake could achieve efficient transfection of non-dividing cells. The involvement of nuclear localization signals only marginally improved transfection over the last decades [70] and could be enhanced by NPC targeting protein domains mimicking the efficient direct nuclear entry of viruses [[71], [72], [73], [74], [75]]. Also, the employment of motor proteins could guide the cytoplasmic DNA towards the nuclear pores which was recently assessed in our group [10]. This study assessed the substitution of VRK1 with dynein motor complex proteins and nuclear proteins to the TFAMoplex. The presence of the dynein light chain protein RP3 alone did not increase the interaction with nuclear proteins, nor the transfection efficiency. Interestingly, the incorporation of RP3 and the nuclear protein leucine-rich repeat-containing protein 59 slightly surpassed the original TFAMoplex in number of transfected cells. In a next step, these proteins could be combined with the SpyTFAMoplex. However, progress in this area is significantly hindered by the lack of direct methodologies to dissect nuclear import routes [62], whether interphase- or mitosis-dependent, which remains a major obstacle to the rational design of specific nuclear delivery mechanisms for non-viral vectors. Collectively, our findings support that active enzymes can be co-delivered with DNA to modulate its intracellular fate, representing a critical step toward the advancement of efficient non-viral DNA delivery systems.

## 4 Experimental section

### 4.1 Chemicals and consumables

T4 ligase was purchased from New England Biolabs (Ipswich, MA, USA). LB broth was obtained from LLG Labware (Meckenheim, Germany). Isopropyl-β-D-thiogalactopyranosid (IPTG), dithiothreitol (DTT), TRIS-acetate-EDTA (TAE) buffer (50x), and lysozyme were obtained from AppliChem GmbH (Darmstadt, Germany). Fast Digest restriction enzymes, Fast Digest Green Buffer (10x), GeneRuler DNA Ladder Mix, imidazole, FBS, penicillin-streptomycin (10,000 U/mL), blasticidin S hydrochloride (from *Streptomyces griseochromogenes*), trypsin-EDTA (0.25 %), Lipofectamine 3000™, UltraPure agarose, Coomassie Brilliant blue G-250, Pierce™ streptavidin magnetic beads, Dulbecco's Modified Eagle Medium (DMEM) high glucose GlutaMAX supplement, FluoroBrite DMEM, OptiMEM, phosphate buffered saline (PBS) (KCl 2.7 mM, 137 mM, KHPO_4_ 1.8 mM, Na_2_PO_4_ 10.1 mM, pH 7.4), bromophenol blue, and Hoechst 33342, LysoTracker™ DeepRed were obtained from Thermo Fisher Scientific (Waltham, MA, USA). GelRed DNA dye was purchased from Biotium (Hayward, CA, USA). Nickel-nitrilotriacetic acid (Ni-NTA) agarose and QIAprep Spin Miniprep Kit were obtained from Qiagen (Germantown, MD, USA). Syringe filters (0.22 μm) and 24-well and 96-well tissue culture plates were obtained from Techno Plastic Products (TPP) AG (Trasadingen, Switzerland). Protino Columns 14 mL and NucleoBond XtraMidi columns were purchased from MACHERY-NAGEL (Düren, Germany). Fluorofurimazine was purchased from Aobius Inc. (Gloucester, MA, USA). 96-well U-shaped, sterile polystyrene microplates were purchased from Greiner bio-one (Kremsmünster, Austria). EBFP2-N1 plasmid from Addgene #54595, pmScarlet_C1 plasmid from Addgene #85042. Cy3 labeled Plasmid Delivery Control and MFP488 Nucleic Acid Labeling Kit from MirusBio (Madison, WI, USA). Glucose, HEPES, potassium chloride, kanamycin sulfate, protease inhibitor cocktail, poly(ethyleneimine) (PEI) branched, average molecular weight 10,000 g/mol, heparin agarose, ethanol, acetonitrile, formic acid, sodium dodecyl sulfate (SDS), glycerol, 2-mercaptoethanol, Tris hydrochloride (Tris-HCl), and bovine serum albumin (BSA), methanol-free paraformaldehyde (PFA), ZnCl_2_, Chloramphenicol, NaCl, agarose, and all other chemicals, when not otherwise stated, were from Sigma Aldrich Chemie GmbH (Buchs, Switzerland).

### 4.2 Plasmid construction

Cloning was performed according to the manufacturer's protocols for FastDigest restriction enzymes (Thermo Fisher Scientific) and T4 DNA ligase (New England Biolabs). DNA inserts were synthesized by GeneArt services (Thermo Fisher Scientific) and Twist Bioscience (South San Francisco, CA, USA). Plasmids and inserts were digested with the respective restriction enzymes. Next, digested backbones and inserts were ligated and subsequently transformed into *E. coli* DH5α cells (18265017, Thermo Fisher Scientific). Plasmids were purified using the QIAprep Spin Miniprep Kit or NucleoBond XtraMidi columns. Table S4 contains the DNA sequences of all constructs. The TFAM variant containing the two cysteine point mutations A105C and V109C [45] was used in the TFAM-VRK1, TFAM-VRK1-mScarlet, and TFAM-SpyTag constructs. The SpyCatcher-dVRK1 construct was obtained by introducing a dead mutation into the active site of VRK1 (D177A) using site directed mutagenesis. All pET vectors used in this study are derived from the pET His6 TEV LIC cloning vector (1B), developed by Scott Gradia (Addgene plasmid #29653, Addgene, Cambridge, MA, USA).

### 4.3 Protein expression and purification

For the TFAM fusion constructs and SpyCatcher-VRK1, and SpyCatcher-dVRK1, the respective plasmids were transformed into chemically competent *E. coli* BL21(DE3)pLysS (L1195, Promega AG, Dübendorf, Switzerland). The transformed cells were cultured in 700 mL LB medium supplemented with 50 mg/L kanamycin and 0.2 % glucose. The bacteria were grown at 37 °C at 250 rpm until the optical density at 600 nm (OD600) reached 0.5–0.7. Subsequently, protein expression was induced by addition of 0.4 mM IPTG and incubation at 30 °C. After 5 h, the cells were centrifuged (ST16R, Thermo Fisher Scientific) at 5000×*g*, 10 min, 4 °C. The resulting cell pellet was stored overnight at −20 °C.

The next day, the pellet was thawed and resuspended in lysis buffer (1x PBS pH 7.4, KCl (1 M), DTT (1 mM), lysozyme (1 mg/mL), 1x protease inhibitor cocktail) and subsequently lysed by sonication on ice. Next, 0.1 % PEI was added to precipitate nucleic acids, and the lysate was centrifuged (30,000×*g*, 45 min, 4 °C) using a Sorvall LYNX 6000 centrifuge (Thermo Fisher Scientific) to remove cell debris and DNA. The supernatant was filtered with a 0.22 μm syringe filter, supplemented with 10 mM imidazole, and loaded onto a 1 mL Ni-NTA agarose column. Washing was performed with 10 column volumes (CV) of wash buffer (1x PBS pH 7.4, KCl (1 M), DTT (1 mM), imidazole (25 mM)), and proteins were eluted with elution buffer (1x PBS pH 7.4, KCl (1 M), DTT (1 mM), imidazole (250 mM). Protein-containing fractions were pooled and diluted seven-fold with cold MilliQ to reduce the salt concentration to below 180 mM. Next, the diluted solution was immediately applied onto a 1 mL heparin-agarose column to remove bacterial DNA. Washing was performed with 5 CV of wash buffer (1x PBS pH 7.4, DTT (1 mM)). The protein was eluted with elution buffer (1x PBS pH 7.4 and 1 M KCl). Finally, buffer exchange was performed to storage buffer (0.5x PBS pH 7.4, 10 % glycerol) using 10,000 or 30,000 MWCO Amicon Ultra Centrifugal Filters (Sigma Aldrich). The protein was concentrated to approximately 1 mg/mL, aliquoted, and snap-frozen in liquid nitrogen and stored at −80 °C. Protein concentration was determined spectrophotometrically at 280 nm using a NanoPhotometer Pearl (Implen GmbH, Munich, Germany).

PLC-TFAM and a phospholipase-dead construct (dPLC-TFAM) having a tryptophan to glycine mutation on active site residue 52 (W52G) were produced with a maltose binding protein (MBP) as solubility tag (MBP-PLC-TFAM). The MBP was connected to the N-terminus of PLC with a flexible linker and a tobacco etch virus (TEV) protease cleavage site (Table S4). The N-terminal addition keeps the PLC inactive during production, as the N-terminal residue is part of the catalytic center. TEV protease was used to cleave off MBP *in vitro*. MBP-PLC-TFAM was expressed as described above. Production and purification was performed as described previously [45]. In short, the thawed bacterial cell pellet was resuspended in lysis buffer (0.5x PBS, glycerol (10 %), KCl (500 mM), DTT (1 mM), and incubated for 30 min on ice followed by lysis via sonication. Next, KCl (500 mM) and final 0.1 % PEI were added and the lysate was centrifuged at 30,000×*g* for 45 min at 4 °C. The supernatant was filtered with a 0.22 μm syringe filter, supplemented with 10 mM imidazole before application onto a 1 mL Ni-NTA agarose column. The column was washed with 15 CV of wash buffer (0.5x PBS, glycerol (10 %), KCl (500 mM), imidazole (20 mM), DTT (1 mM)) and eluted with elution buffer (wash buffer supplemented with final 250 mM imidazole). The eluted protein was incubated with TEV protease in a 1:10 w/w ratio and incubated overnight at 4 °C. The next day, the sample was purified with a heparin column as described above to remove cleaved MBP and TEV protease. Buffer exchange was performed as described above with storage buffer consisting of 0.5x PBS supplemented with 20 % glycerol and 5 mM MgCl_2_. The final protein was concentrated to approx. 1 mg/mL, aliquoted, snap-frozen in liquid nitrogen, and stored at −80 °C. Protein size and purity were estimated by sodium dodecyl sulfate polyacrylamide gel electrophoresis (SDS-PAGE) and densitometric analysis using the software ImageJ [76].

The BAF protein used for gel mobility shift assays was produced as described in reference [34]. His_6_-MBP-BAF was expressed in the inactive hyperphosphorylated state together with VRK1 in *E. coli* BL21(DE3)pLysS at 37 °C for 3 h, centrifuged (4 °C, 4000×*g*, 10 min) and frozen at −20 °C. The next day, the thawed cell pellet was resuspended in lysis buffer (HEPES (20 mM, pH 7.4), KCl (150 mM), glycerol (10 %), MgCl_2_ (10 mM)) supplemented with protease inhibitor cocktail and lysozyme (1 mg/mL). After sonication, the lysate was centrifuged (20,000×*g*, 4 °C, 45 min). The sterile filtered supernatant was loaded onto a Ni–NTA column that was equilibrated with lysis buffer supplemented with imidazole (10 mM). After washing with lysis buffer containing 10 mM imidazole, His_6_-MBP-BAF was eluted in lysis buffer with 250 mM imidazole. In parallel, lambda phosphatase was prepared with the same protocol but with a modified lysis buffer (HEPES (50 mM, pH 7.5), NaCl (0.2 M), imidazole (5 mM), glycerol (10 %), MnCl_2_ (1 mM)). For BAF dephosphorylation and His_6_-MBP cleavage, BAF was mixed in a 1:1 w/w ratio with the lambda phosphatase, and 1:100 (w/w) with TEV protease and incubated overnight at 30 °C in HEPES (20 mM, pH 7.5) with NaCl (1 M), glycerol (10 %), MnCl_2_ (1 mM), and DTT (2 mM). The next day, the buffer was exchanged into lysis buffer. The cleaved protein mixture was supplemented with 20 mM imidazole and loaded again onto a Ni-NTA column, but the flow-through was collected containing the dephosphorylated, purified BAF without His_6_-MBP. Final protein purification was performed with Superdex 75 10/300 GL column (GE Healthcare, Chicago, IL USA) with a flow rate of 0.4 mL/min in running buffer (HEPES (20 mM, pH 7.4), KCl (150 mM), glycerol (10 %)). Purified wild-type BAF was snap-frozen in liquid nitrogen.

### 4.4 SDS-PAGE

One μg of protein was mixed with Lämmli buffer (BioRad, Hercules, CA, USA), boiled at 95 °C for 5 min and loaded on the gel. In addition to assessing the protein size and purity, the coupling reaction of TFAM-SpyTag with SpyCatcher-VRK1 and SpyCatcher-dVRK1 was also confirmed by SDS-PAGE analysis. Therefore, one μg of TFAM-SpyTag was incubated for 15 min at room temperature (RT) with one μg of the respective coupling reaction partner and subsequently mixed with Lämmli buffer, boiled at 95 °C for 5 min and loaded on the gel. The samples were loaded onto precast 12-well gels (BioRad) and electrophoresis was performed at 100–120 mA for approx. 90 min in SDS-PAGE running buffer (25 mM Tris-HCl, 200 mM glycine, 0.1 % SDS, pH 8). Gels were stained with Coomassie solution (0.1 % Coomassie Brilliant Blue, 10 % acetic acid, 30 % methanol) for 30 min and destained (10 % acetic acid, 30 % methanol). Finally, images were captured using a ChemiDoc system (BioRad). The resulting images are illustrated in Fig. S3.

### *4.5* *In vitro* VRK1 kinase activity assay (gel mobility shift)

Agarose gels were prepared by using 0.8 %–1.0 % (w/v) agarose in TAE buffer and GelRed DNA dye (1:40,000). First, TFAM-SpyTag was mixed with SpyCatcher-VRK1 or with SpyCatcher-dVRK1 at 0, 0.1 or 0.25 μM final concentrations for each protein and incubated for 15 min at RT. Second, 1x T4 DNA Ligase Buffer (New England Biolabs) containing ATP for kinase activity, was added to a final volume of 10 μL. Third, mScarlet-pDNA was added with a final concentration of 10 ng DNA/μL and the mixture was further incubated for 20 min for particle formation. Lastly, BAF at 0 or 1 μM was added and again incubated for 20–30 min. Two μL of Fast Digest Green Buffer (10x) were added and 5–10 μL of the mixture were transferred into one well of the agarose gel. The gel was run for about 45 min at 100 mA in an electrophoresis chamber (Sub-Cell GT Cell, Bio-Rad). The DNA was imaged with a ChemiDoc MP Gel reader (Bio-Rad).

### 4.6 Dynamic light scattering (DLS) experiments

Particle formation was performed with 0.6 μM TFAM-SpyTag, 0.4 μM SpyCatcher-VRK1 or SpyCatcher-dVRK1, 10 ng mScarlet-pDNA/μL at RT for 20 min in a total volume of 20 μL using 1x PBS, pH 7.4 as buffer. Before DLS measurement, 40 μL PBS were added and then 50 μL of the dilution were transferred to a micro cuvette (ZEN0040, Malvern Panalytical, Malvern, UK) for measurement. The hydrodynamic diameter was measured based on the intensity of the scattered light using a Zetasizer pro (Malvern Panalytical). For size determination, the average peak values of 3 independent experiments, performed in technical triplicates, were taken.

### 4.7 Cell culture

HeLa (ATCC CCL-2, human, female) cells were purchased from ATCC (Manassas, VA, USA). HeLa cells stably overexpressing EGFP-BAF (EGFP-BAF cells) were a kind gift from Prof. Dr. Gerlich (IMBA, Vienna, Austria) [26]. All HeLa cell lines (passage number 3–30) were maintained in medium (DMEM GlutaMAX™, 10 % FBS, 1 % (v/v) pen-strep) at standard cell culture conditions (37 °C, 5 % CO_2_, humidified atmosphere). EGFP-BAF cells were cultured in complete growth medium with 6 μg/mL blasticidin. Cells were tested negative for mycoplasma contamination (MycoAlert Kit, Lonza AG, Basel, Switzerland).

### 4.8 Cell preparation for transfection efficiency analysis

In 24-well tissue culture plates 80,000 HeLa cells were seeded per well to reach 90 % confluency on the day of transfection. Before transfection, the cells were washed once with PBS and 500 μL of fresh medium were added to the cells.

### 4.9 Particle formation and transfection

Original TFAMoplex formation and Lipofectamine-based transfection were performed as described previously [53]. SpyTFAMoplex and dSpyTFAMoplex formation were performed in ≥80 % FBS in 1.5 mL test tubes with a final volume of 10–100 μL per protein. If not indicated otherwise, TFAM-SpyTag (1 μM) and SpyCatcher-VRK1 (1 μM) or SpyCatcher-dVRK1 (1 μM) were mixed and incubated for 15 min at RT. Then, FBS was added followed by PLC-TFAM (1 μM) and pDNA with a final concentration of 10 ng pDNA/μL. The samples were mixed by tipping and incubated at RT for 20 min to allow protein-DNA complex formation before transferring 5–20 μL of the mixture to cells.

### 4.10 Widefield microscopy

To visualize the reporter gene expression one day after transfection at 200 ng mScarlet-pDNA/mL medium for 30 min, transfected HeLa cells in 24-well plate wells were washed with 500 μL PBS and then transferred to a Leica DMi6000 Inverted Fluorescence Microscope (Leica Microsystems, Wetzlar, Germany). Cells in PBS were imaged at 10x magnification. Brightfield images were acquired as well as mScarlet fluorescence with an excitation filter of 542–582 nm and an emission filter of 604–644 nm.

### 4.11 Flow cytometry

Transfection efficiency was analyzed one day after transfection at 200 ng mScarlet-pDNA/mL medium for 30 min. The day of analysis, HeLa cells were washed three times with PBS, trypsinized for 3 min at 37 °C and transferred to a 96-well U-shaped microplate containing 20 μL FBS. Next, the cells were centrifuged at 300×*g*, 3 min, 4 °C (Sorvall ST 16R centrifuge, Thermo Fisher Scientific) followed by resuspending the pellets in ice cold flow cytometry buffer (PBS, 1 mM EDTA, 1 % BSA) and flow cytometry analysis (CytoFLEX Flow Cytometer, Beckman Coulter Life Sciences, Nyon, Switzerland). For each sample, 10,000 cells were analyzed according to their fluorescence (mScarlet) using 561 nm excitation with a bandwidth of 10 nm and recorded with a 585 nm emission filter with a bandwidth of 42 nm using the FlowJo software (Tree Star Inc., Ashland, OR, USA). The measurements were analyzed with the FlowJo software (Tree Star Inc.). 1 % of the untreated cells were set as mScarlet-positive. The gating was subsequently applied to all samples. Each condition was measured in 6 independent experiments performed in technical triplicates.

### 4.12 Cytotoxicity/cell viability assay

HeLa cells were seeded in a 96-well tissue culture plate at 5000 cells per well in 100 μL medium. The next day, cells were washed once with 100 μL PBS, fresh medium was added and the cells were transfected with SpyTFAMoplexes, dSpyTFAMoplexes or Lipofectamine with 400, 200, 40, or 4 ng mScarlet-pDNA/mL. As controls, proteins only (same concentration as the 400 ng pDNA/mL condition), 400 ng pDNA/mL, or SDS at a final concentration of 2 % (w/v) were used. After one day of incubation, the medium in each well was changed to 100 μL Fluorobrite DMEM with 20 % (v/v) CellTiter 96 Aqueous One Solution Reagent (Promega Corporation, Madison, WI, USA) without washing step. The cells were incubated for 1.5 h under standard cell culture conditions (37 °C, 5 % CO_2_, humidified atmosphere) followed by measuring absorbance at 490 nm with a Spark Multimode Microplate reader (Tecan, Männerdorf, Switzerland). Data were normalized to the non-treated control and absorbance value obtained for the positive control (SDS) was subtracted from each sample. Measurement was performed in three biological replicates each with three technical replicates.

### 4.13 Cell preparation for confocal microscopy

For confocal microscopy experiments, 20,000–30,000 EGFP-BAF cells were seeded per 1 cm^2^ μ-slide well (μ-slide 8 well chambered coverslips, glass bottom, ibidi, Martinsried, Germany) with a working volume of 250 μL. The next day, the cells were washed once with PBS and 250 μL fresh medium or 250 μL of 100 % FBS were added to the cells to prepare them for transfection experiments. If not indicated otherwise, transfection was performed with 400 ng DNA/mL. If not indicated otherwise, the cells were washed three times with PBS after 30 min incubation and further incubated with the transfection agents in medium until the respective timepoint, *i.e.* 3 h to assess early BAF clustering, 4 h for advanced BAF clustering, or overnight. Before imaging, the cells were washed three times with PBS, eventually fixed with 4 % methanol-free PFA for 20 min and optionally stained with 2.5 μg/mL Hoechst 33342 for 20 min. After staining, the cells were washed three times with PBS and finally, 250 μL PBS were added to the cells for imaging and storage. Fixed microscopy samples were stored at 4 °C and imaged within one week.

### 4.14 Confocal laser microscopy

Confocal laser microscopy was performed on an Eclipse Ti2 inverse spinning disk confocal microscope (Nikon, Tokyo, Japan) with a dual disk technology of 50 μm pinholes at 500 μm spacing. For acquisition, a 100x 1.45 CFI Plan Apo Oil objective (Nikon) with immersion oil type F (Nikon) or a 40x 1.41 Plan Apo Lambda Silicon oil objective (Nikon) with silicon oil (Nikon) and a sCMOS Orca Fusion BT camera (2304 x 2304 pixel, 6.5 μm x 6.5 μm pixel size) (Hamamatsu Photonics, Hamamatsu City, Japan) were used. The imaging conditions were set to the following: The Hoechst and EBFP fluorophores were excited at 405 nm and emission was collected with a 447 nm filter (bandwidth 60 nm). Green fluorophores were excited at 488 nm and detected using a 525 nm filter (bandwidth 50 nm). Cy3, AF594, or mScarlet were excited at 561 nm and fluorescence of these dyes was detected with a 600 nm filter (bandwidth 52 nm). Cy5 dyes (Lysotracker DeepRed, AF647) were excited at 647 nm and emission was collected with a 708 nm filter (bandwidth 75 nm). If not indicated otherwise, fluorophores were excited at 20 % laser intensity and 20 ms exposure time and 35 z-stacks were acquired with a slice thickness of 0.3 μm with the 100x objective and 31 z-stacks of 0.5 μm with the 40x objective. The software ImageJ [76] was used for image analysis.

### 4.15 Quantitative EGFP-BAF cells and cluster segmentation

To quantitatively characterize clustering of pDNA by BAF in EGFP-BAF cells, we adapted an image analysis and data processing workflow previously described in reference [53]. For image analysis, z-projections of maximum intensity of 31 z-stacks with 0.5 μm stack slice acquired with the 40x objective were used and processed with the ImageJ Macro 1 EGFP-BAF cells and clusters (Table S2).

Cells were counted via segmentation in the EGFP-BAF channel by intensity thresholding (280 to 65535) with a size of 200 μm^2^ to infinity and a circularity of 0.4 to 1.0, if not indicated otherwise. Reporter gene expression intensity was measured in the cells. The number of cells were visually checked and corrected when necessary. EGFP-BAF clusters were segmented by intensity thresholding (2500 to 65535) with a size from 0.02 to 200 μm^2^ and with a circularity of 0.8 to 1.0. Area, spatial location (xy coordinates of the centroid), and MFI in the segmented EGFP-BAF clusters were measured. All data in csv format were transferred via MATLAB to the Excel template: *seg EGFP_BAF_cells_clusters_RepGene express_template*. Further thresholding in cluster size was performed within the Excel template considering only the cluster ROIs larger than the apparent area of 0.05 μm^2^. Four biological replicates were performed. In each biological replicate, 3 to 9 images were used per transfection condition.

### 4.16 Timelapse imaging

For timelapse live-cell imaging in 1 cm^2^ μ-slide wells, 20,000 EGFP-BAF cells were seeded one day before imaging. Before treatment, they were washed once with PBS, followed by the addition of FluoroBrite DMEM supplemented with 10 % FBS and 1 % PenStrep (imaging medium). Transfection was performed in imaging medium and imaging was performed without further washing of the cells at 37 °C and 5 % CO_2_. If not indicated otherwise, 17 z-stacks with a slice thickness of 0.5 μm using the 40x objective were acquired every 2–10 min for 4 to 15 h. EGFP was excited at 488 nm and detected with a 525 nm filter (bandwidth 50 nm). Cy3 and mScarlet were excited at 561 nm and detected with a 600 nm filter (bandwidth 52 nm). Unless stated otherwise, imaging conditions were 5 % laser intensity and 100 ms exposure time for all applied channels.

### 4.17 Anti-VRK1 AB staining

EGFP-BAF cells (70,000) were seeded on coverslips (Marienfeld, Germany) in 24-well plate wells, transfected the next day and fixed 4 h after transfection with 4 % methanol-free PFA for 20 min. Subsequently, they were permeabilized with 0.1 % TritonX-100 for 5 min. Blocking was performed with 2 % BSA in PBS for 1 h at RT. The primary AB (mouse anti-VRK1 AB iGG1 AB171933 abcam) was applied as 1:200 dilution in PBS (5 μg/mL) for 1.5 h at RT. The secondary AB (goat anti-mouse IgG H&L, AF594, AB150116, abcam) was applied as 1:1000 dilution (2 μg/mL) in PBS with 1 % BSA for 1 h at RT. For mounting, ProLong^TM^ Diamond Antifade Mountant (Thermo Fisher Scientific) was used. In between the steps, cells were washed three times with 500 μL PBS. Samples were stored at 4 °C.

### 4.18 Anti-emerin AB staining

EGFP-BAF cells (70,000) were seeded on coverslips in 24-well plate wells. The next day, they were transfected and washed three times with 500 μL PBS 30 min after transfection and further incubated in medium for a total of 4 h or 24 h after transfection. According to the timepoint, the cells were washed three times with 500 μL PBS and fixed with 4 % methanol-free PFA for 20 min. The cells were permeabilized with 0.1 % Triton X-100 for 5 min and blocked with 2 % BSA in PBS for 1 h at RT. The primary AB (mouse anti-emerin AB sc-398278, Santa Cruz Biotechnology, Dallas, TX, USA) was diluted in 1 % BSA in PBS to a final concentration of 2 μg/mL, in 200 μL per well, and incubated for 90 min at RT. The secondary antibody (goat anti-mouse IgG H&L, AF594, AB150116, abcam) was diluted to 2 μg/mL in 1 % BSA in PBS and applied in 200 μL per well for 1 h at RT. Finally, the cells were mounted with ProLong™ Diamond Antifade Mountant. Between each step, cells were washed three times with 500 μL PBS. To determine AF594 background signal, samples with transfected EGFP-BAF cells were mounted after the blocking and wash step, but not immunostained. Mounted cells were stored at 4 °C until imaging.

### 4.19 Quantitative colocalization analysis EGFP-BAF cluster and anti-emerin AB AF594

To quantitatively characterize recruitment of emerin to BAF clusters in transfected EGFP-BAF cells, we applied an image analysis and data processing workflow similar to section 4.15. For image analysis, z-projections of maximum intensity of 38 z-stacks with 0.2 μm slice thickness acquired with the 100x objective were used and processed with the ImageJ Macro 2 EGFP-BAF cells and Macro 3 EGFP-BAF clusters and emerin (Table S2). Cells were counted via segmentation in the EGFP-BAF channel by setting an intensity threshold from 130 to 65535, an object size from 200 μm^2^ to infinity, and a circularity from 0.4 to 1.0. The number of cells were visually checked and corrected when necessary. Similarly, EGFP-BAF clusters were segmented by intensity thresholding (350 to 65535) with a size from 0.05 to 100 μm^2^ and a circularity of 0.95 to 1.0. The MFI of the segmented EGFP-BAF clusters were measured in the AF594 and EGFP-BAF channels. Segmented EGFP-BAF clusters were visually checked in the EGFP channel and signals not referring to cluster shapes, such as high-intensity signals referring to the nuclear rim, were excluded. Image analysis was performed with 3–6 images per condition, including non-immunostained controls, in one biological replicate.

To determine a threshold whether an EGFP-BAF cluster is emerin-positive or -negative, z-projections of non-immunostained but transfected EGFP-BAF cells were used (Fig. S10). Therefore, the average MFI in the AF594 channel plus 1.5 times the SD of all segmented and visually confirmed EGFP-BAF clusters in all transfection conditions was set as background MFI threshold (160.81) (Fig. S10B). Consequently, in immunostained samples, clusters with an AF594 signal >160.81 were considered emerin-positive (Fig. S11).

### 4.20 Anti-Cy3 AB647 counterstaining

EGFP-BAF cells (30,000) were seeded per 1 cm^2^ μ-slide well. The next day, they were transfected with 400 ng Cy3-DNA/mL medium and washed three times with 250 μL PBS and further incubated in medium. After a total of 4 or 24 h after transfection, the cells were washed three times with 250 μL PBS and fixed with 4 % methanol-free PFA for 20 min. After fixation, the cells were washed three times with 250 μL PBS and incubated for 20 min with 200 μL of 0.8 μg/mL anti-Cy3 AB647 (sc-166894, Santa Cruz Biotechnology) diluted in PBS. Then, the cells were washed three times with 250 μL PBS and stored in 250 μL PBS at 4 °C until imaging.

Sample excitation on the confocal microscope with the 100x objective was performed at 488 nm, 561 nm, and 647 nm, all excited for 200 ms at 20 % laser intensity. Thirty-eight slices with a thickness of 0.2 μm were acquired per image. Images were acquired in 3 independent experiments, with 3–6 images per condition per experiment, including untreated controls.

### 4.21 MFP488-labeled pDNA colocalization with Lysotracker DeepRed

EBFP-pDNA was labeled at a 0.1 % (w/v) DNA to labeling reagent ratio and purified with ethanol precipitation according to the manufacturer's protocol (MirusBio). HeLa cells (35,000) were seeded per 1 cm^2^ μ-slide well. The next day, the cells were transfected as described above with 400 ng MFP488-labeled pDNA/mL medium, washed after 30 min three times with 250 μL PBS and further incubated in medium. After a total of 4 h or one day after transfection, cells were washed three times with 250 μL PBS, and Lysotracker DeepRed (0.25 μM, 1:4000 dilution) was incubated with the cells in imaging medium for 15 min at standard cultivation conditions. After washing three times with 250 μL PBS, fresh imaging medium was given to the cells and image acquisition (26 stacks, 0.3 μm slice thickness) was immediately performed with living cells. Excitation was performed at 647 nm (1 % laser intensity, 50 ms) and 488 nm (20 % laser intensity, 200 ms).

### 4.22 Proteomics

#### 4.22.1 Sample preparation

The proteome and phosphopeptide quantification and identification was performed in collaboration with the Functional Genomics Center Zürich (FGCZ). 1 × 10^6^ HeLa cells were treated with SpyTFAMoplex or dSpyTFAMoplex with 400 ng mScarlet-pDNA/mL medium. After 4 h incubation, cells were washed three times with 100 μL PBS, trypsinized for 5 min at 37 °C and transferred to a centrifuge tube containing 100 μL FBS. The cells were washed three times with 1 mL PBS by centrifugation (500×*g*, 5 min) and the dry cell pellet was snap frozen in liquid nitrogen and stored at −80 °C until analysis at FGCZ.

#### 4.22.2 Protein extraction

To lyse the samples, 100 μL of lysis buffer containing 2 % SDS, 100 mM Tris/HCL pH 8.2 were added and the samples were treated with High Intensity Focused Ultrasound (HIFU) for 1 min at an ultrasonic amplitude of 90 %. The samples were boiled for 5 min at 95 °C and the supernatant was recovered by centrifugation for 10 min at 20,000×*g*. The supernatant was treated with 5 Units of Benzonase (Sigma Aldrich) per sample for 30 min at 37 °C in a Thermoshaker at 700 rpm. The protein concentration was determined using the Lunatic UV/Vis polychromatic spectrophotometer (Unchained Labs, Pleasanton, CA, USA) with a 1:10 (v/v) dilution for each sample.

#### 4.22.3 Protein digestion

For each sample 80 μg of protein, according to Lunatic measurement, were taken and reduced with 5 mM tris(2-carboxyethyl)phosphine (TCEP) and alkylated with 15 mM chloroacetamide at 30 °C for 30 min in the dark. Samples were processed using the single‐pot solid‐phase enhanced sample preparation (SP3). The SP3 protein purification, digest and peptide clean-up were performed using a KingFisher Flex System (Thermo Fisher Scientific) and Carboxylate-Modified Magnetic Particles (Sigma Aldrich) [77]. Beads were conditioned following the manufacturer's instructions, consisting of 3 washes with water at a concentration of 1 μg/μL. Samples were diluted (v/v) with 100 % ethanol to a final concentration of 60 % ethanol. The beads, wash solutions and samples were loaded into 96 deep well- or micro-plates and transferred to the KingFisher. Following steps were carried out on the robot: collection of beads from the last wash, protein binding to beads, washing of beads in wash solutions 1–3 (80 % ethanol), protein digestion (overnight at 37 °C with a chymotrypsin:protein ratio (w/w) of 1:50 in 50 mM Triethylammoniumbicarbonate (TEAB)) and peptide elution from the magnetic beads using MilliQ water. The digest solution and water elution were combined and dried to completeness.

#### 4.22.4 Phosphopeptide enrichment

The phosphopeptide enrichment was performed using a KingFisher Flex System (Thermo Fisher Scientific) and Ti-IMAC HP MagBeads (ReSyn Biosciences, Pretoria, South Africa). Beads were conditioned following the manufacturer's instructions, consisting of three washes with 400 μL of binding buffer (0.1 M glycolic acid, 80 % acetonitrile, 5 % trifluoroacetic acid (TFA)). Each fraction was dissolved in 150 μL binding buffer and an aliquot corresponding to 1 μg per sample was kept for whole proteome analysis. Beads, wash solutions and samples were loaded into 96 well microplates and transferred to the KingFisher. Phosphopeptide enrichment was carried out using the following steps: binding of the phosphopeptides to the beads (30 min), washing the beads in wash 1, 2 and 3 (wash buffer 1: 0.1 M glycolic acid, 80 % acetonitrile, 5 % TFA; wash buffer 2: 80 % acetonitrile, 1 % TFA; wash buffer 3: 10 % acetonitrile, 0.2 % TFA, 3 min each) and eluting peptides from the beads (80 μL 1 % NH_4_OH in water, 10 min). To each elution 10 μL of 20 % formic acid were added. Phospho-enriched as well as 20 % of the small input fraction for full proteome analysis were loaded onto Evotips, according to the manufacturer's instructions.

#### 4.22.5 Liquid chromatography (LS) - mass spectrometry (MS) analysis

MS analyses were performed on a timsTOF Pro (Bruker, Billerica, MA, USA) coupled to an Evosep One (EvoSep Biosystems, Odense, Denmark). Samples were separated with the extended Evosep method: 15 samples/day, keeping the analytical column (PepSep, ReproSil C18 15 cm x 150 μm, 1.5 μm) at 50 °C. For the dual timsTOF, MS spectra were scanned from *m/z* 100 to *m/z* 1700 in data dependent acquisition Parallel Accumulation Serial Fragmentation (ddaPASEF) mode. For the ion mobility settings, the inversed mobilities (1/K0) from 0.60 Vs/cm^2^ to 1.60 Vs/cm^2^ were analyzed with ion accumulation and ramp time of 100 ms, respectively. One survey TIMS-MS scan was followed by 10 PASEF ramps for MS/MS acquisition, resulting in a 1.17 s cycle time. Singly charged ions were excluded using the polygon filter mask and isolation windows for MS/MS were set to *m/z* 2.0 for precursor ions below *m/z* 700, and *m/z* 3.0 for ions above. The mass spectrometry proteomics data were handled using the local laboratory information management system (LIMS) and all relevant data are deposited to the ETH Research Collection.

#### 4.22.6 Protein and phosphopeptide identification and label free quantification

Individual data analysis workflows have been used for global protein and phosphopeptide analysis. The acquired shotgun MS data were processed for identification and quantification using Fragpipe 22.0. Spectra were searched against a Uniprot Homo sapiens reference proteome (UP000005640, reviewed canonical version from 2025–03–26 concatenated to its reversed fasta entries and common protein contaminants) using MSFragger 4.1 and Percolator. Carbamidomethylation of cysteine was set as fixed modification, while methionine oxidation (and phospho STY for enriched samples) was set as variable. Enzyme specificity was set to chymotrypsin/P allowing a minimal peptide length of 6 amino acids and a maximum of two missed cleavages. Label free quantification and match between run options were enabled. Default Philosopher (version 5.1.1) ion, peptide and protein false discover rate (FDR) values (0.01 %, 1 %, 1 %) were used and phosphosite re-scoring was performed using the integrated ptmProphet node with default settings. MS1 quantification was performed by IonQuant (version 1.10.27) with MaxLFQ activated.

#### 4.22.7 Differential expression analysis

The R package prolfqua [78] was used to analyze the differential expression and to determine group differences (log_2_ fold-changes), confidence intervals, and FDRs (adjusted p-values using Benjamini-Hochberg method) for all quantifiable phosphopeptides and the proteins. In the case of the phosphopeptide analysis it was started with the *combined_site_STY_79.9663.tsv* file generated by FragPipe, which reports the precursor ion intensity for each raw file as well as the localization probability. For the analysis, the prolfquapp [79], prophosqua [80] and prolfquappPTMreaders [81] packages that are extentensions of prolfqua were utilized. These packages wrap around the prolfqua main functions and were designed to specifically analyze and create reports for post-translational modification (ptm) data.

In brief, before fitting the linear models, to determine the group differences, the phosphopeptide abundances were log_2_-transformed and variance-stabilized prior to modeling [82]. Then, linear models were fit and the groups were tested using the prolfqua R-package's contrast API. Rather than imputing missing values, the model adjusts the degrees of freedom based on the number of actual observations per feature, which naturally down-weights features with high missingness and improves the reliability of the fold-change and p-value estimates. Empirical Bayes variance moderation was applied [83] to stabilize variance estimates, particularly when sample size was limited.

For the analysis of the total proteome a similar strategy was used but the main input file was the *msstats_ptm.csv* file. To roll-up peptidoform abundances to protein levels, Tukeys-Median Polish was used to estimate protein abundances. Again, before fitting the linear models, we transformed the protein abundances using the variance stabilizing normalization.

For the combination of the statistical analysis of the total proteome with the phospho-enriched analysis, the prophosqua package was used. The result tables of the total proteome were joined with the phospho-enriched results. Then, the group differences of the phosphopeptides were adjusted for the change that was estimated for the protein and the statistics were recalculated using the procedure suggested by Kohler et al. [84].

### 4.23 Statistical analysis

Statistical analysis was conducted with the GraphPad Prism software version 10.4. If not indicated otherwise, data are represented as mean ± SD of 3–6 independent biological experiments, each performed in technical triplicates (flow cytometry), or 3–9 images per condition. Significance was assessed by Repeated Measures or Ordinary one-way ANOVA with Tukey's multiple comparison test or with Dunnett correction when samples were compared to a control.

## CRediT authorship contribution statement

**Christina Greitens:** Writing – review & editing, Writing – original draft, Visualization, Validation, Supervision, Software, Methodology, Investigation, Formal analysis, Data curation, Conceptualization. **Philip Maurer:** Writing – original draft, Visualization, Validation, Investigation, Formal analysis, Data curation. **Selen Balkan:** Writing – original draft, Visualization, Validation, Software, Methodology, Investigation, Data curation. **Jean-Christophe Leroux:** Writing – review & editing, Supervision, Project administration, Funding acquisition. **Michael Burger:** Writing – review & editing, Validation, Supervision, Project administration, Methodology, Funding acquisition, Formal analysis, Conceptualization.

## Funding

This project has received funding from the 10.13039/501100000781European Research Council (ERC) under the European Union's 10.13039/501100007601Horizon 2020 research and innovation program (grant agreement No 884505).

## Declaration of competing interest

The authors declare that they have no known competing financial interests or personal relationships that could have appeared to influence the work reported in this paper.

## Data Availability

The data that support the ﬁndings of this study are deposited in the ETH research collection.
