# Supplementary material for: VRK1 co-delivery mitigates DNA clustering by BAF in TFAMoplex transfection
Source: Mater Today Bio. 2025 Nov 21;35:102588. doi: 10.1016/j.mtbio.2025.102588 (PMC12718190; doi:10.1016/j.mtbio.2025.102588)
Supplement: Multimedia component 1 [file mmc1.docx]

Supporting Information

# VRK1 co-delivery mitigates DNA clustering by BAF in TFAMoplex transfection

*Christina Greitens^a^, Philip Maurer^a^, Selen Balkan^a^, Jean-Christophe Leroux^a,^*, Michael Burger^a,^**

^a^Institute of Pharmaceutical Sciences, Department of Chemistry and Applied Biosciences, ETH Zurich, Vladimir-Prelog-Weg 3, 8093 Zurich, Switzerland

E-mail: jleroux@ethz.ch

E-mail: michael.burger@pharma.ethz.ch

Table of Contents

TFAM-VRK1-mScarlet partially colocalizes with EGFP-BAF clusters after 24 h 3

*In vitro* TFAM-VRK1 kinase activity assay gel mobility shift 4

SDS-PAGE SpyTag/SpyCatcher linker system and all proteins 5

*In vitro* TFAM-Spy-VRK1 kinase activity assay gel mobility shift 6

Particle size measurement via DLS 7

Cytotoxicity assay 8

EGFP-BAF cells 3 h after transfection with Cy3-DNA at different concentrations 9

EGFP-BAF cells 24 h after transfection with Cy3-DNA at different concentrations 10

Volcano plots phosphorylation enrichment and protein abundance 11

Anti-emerin AB AF594 background in EGFP-BAF clusters 12

EGFP-BAF MFI *vs*. anti-emerin AB AF594 MFI in BAF clusters 13

Lipofectamine and DNA only-treated EGFP-BAF cells counterstained with anti-Cy3 AB 14

Colocalization MFP488-labeled pDNA with Lysotracker DeepRed 15

Tables 17

References 27

## TFAM-VRK1-mScarlet partially colocalizes with EGFP-BAF clusters after 24 h

**
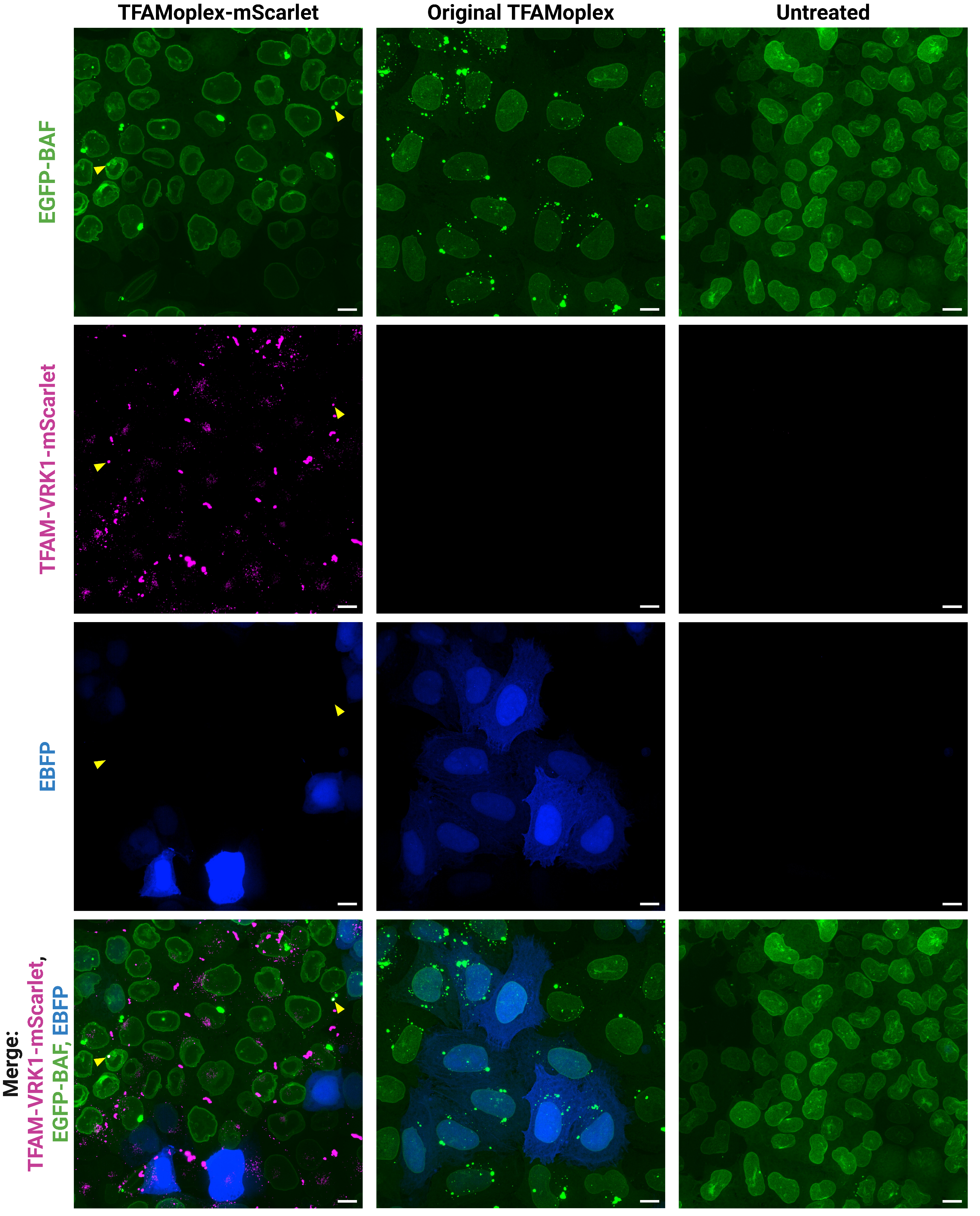
**

**Figure S1**: Colocalization of TFAM-VRK1-mScarlet with EGFP-BAF clusters 24 h after transfection. Confocal microscopy images shown as z-projections of maximum intensities of 35 stacks with 0.3 µm slice thickness. TFAMoplexes were prepared in FBS with PLC-TFAM, TFAM-VRK1-mScarlet or TFAM-VRK1 as control with EBFP-pDNA. EGFP-BAF HeLa cells were transfected for 24 h at 200 ng pDNA/mL medium. Excitation at 20% laser intensity for 200 ms in all channels. Transfected cells were washed, fixed and imaged. Magenta: TFAM-VRK1-mScarlet (intensity: 300-1000). Green: EGFP-BAF (intensity: 50-1000). Blue: EBFP expression (intensity: 150-300). Orange arrowheads indicate colocalization of mScarlet signal with EGFP-BAF clusters. Scale bars: 10 µm.

## *In vitro* TFAM-VRK1 kinase activity assay gel mobility shift


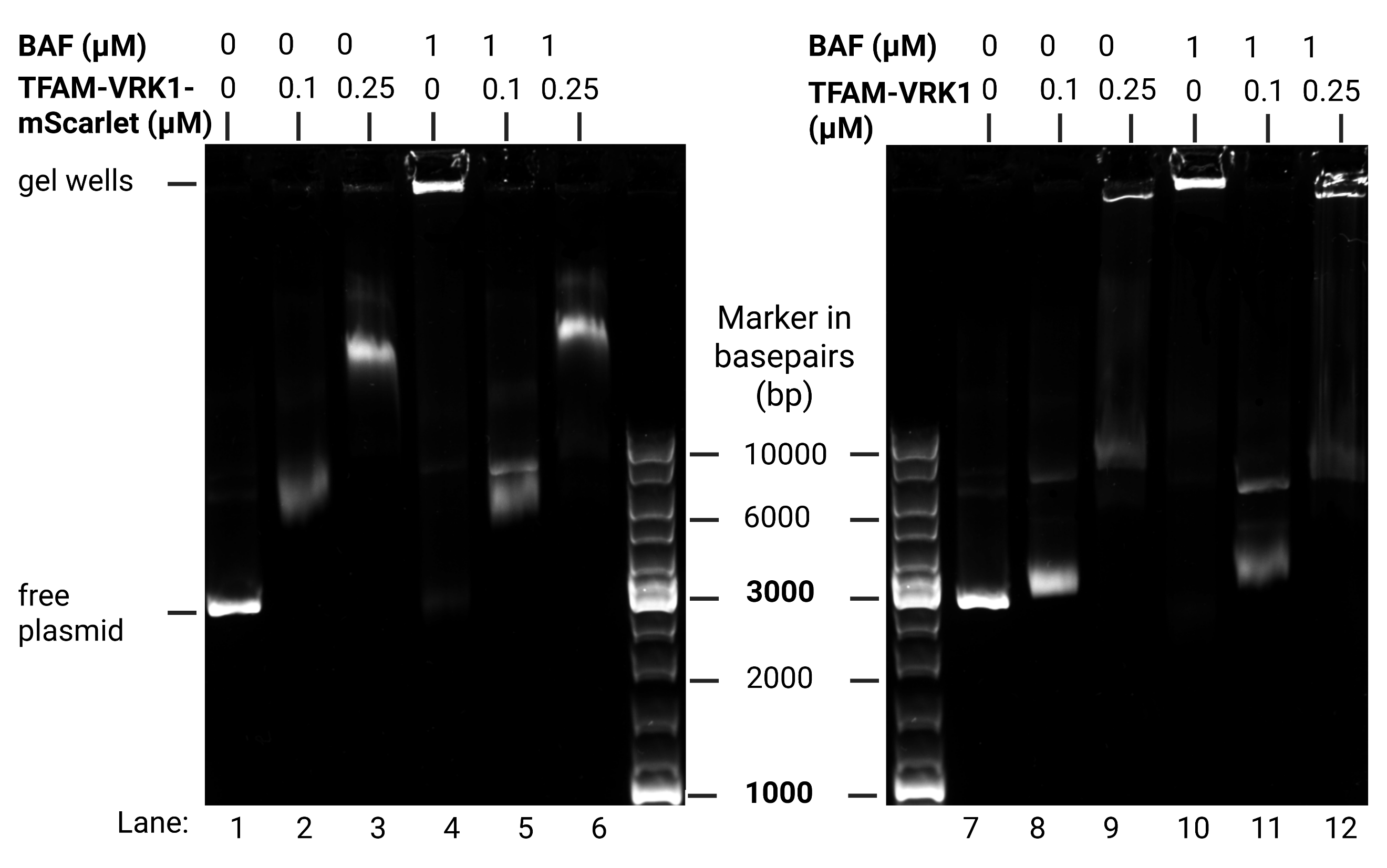


**Figure S2**: Gel mobility shift assay to determine the *in vitro* VRK1 kinase activity. TFAM-VRK1-mScarlet and TFAM-VRK1 were tested at different concentrations (0, 0.1, 0.25 µM). Lanes 1-3 and 7-9 without BAF. Lanes 4-6 and 10-12 with 1 µM BAF. Marker: GeneRuler DNA Ladder Mix. 50 ng pDNA per well.

## SDS-PAGE SpyTag/SpyCatcher linker system and all proteins

**
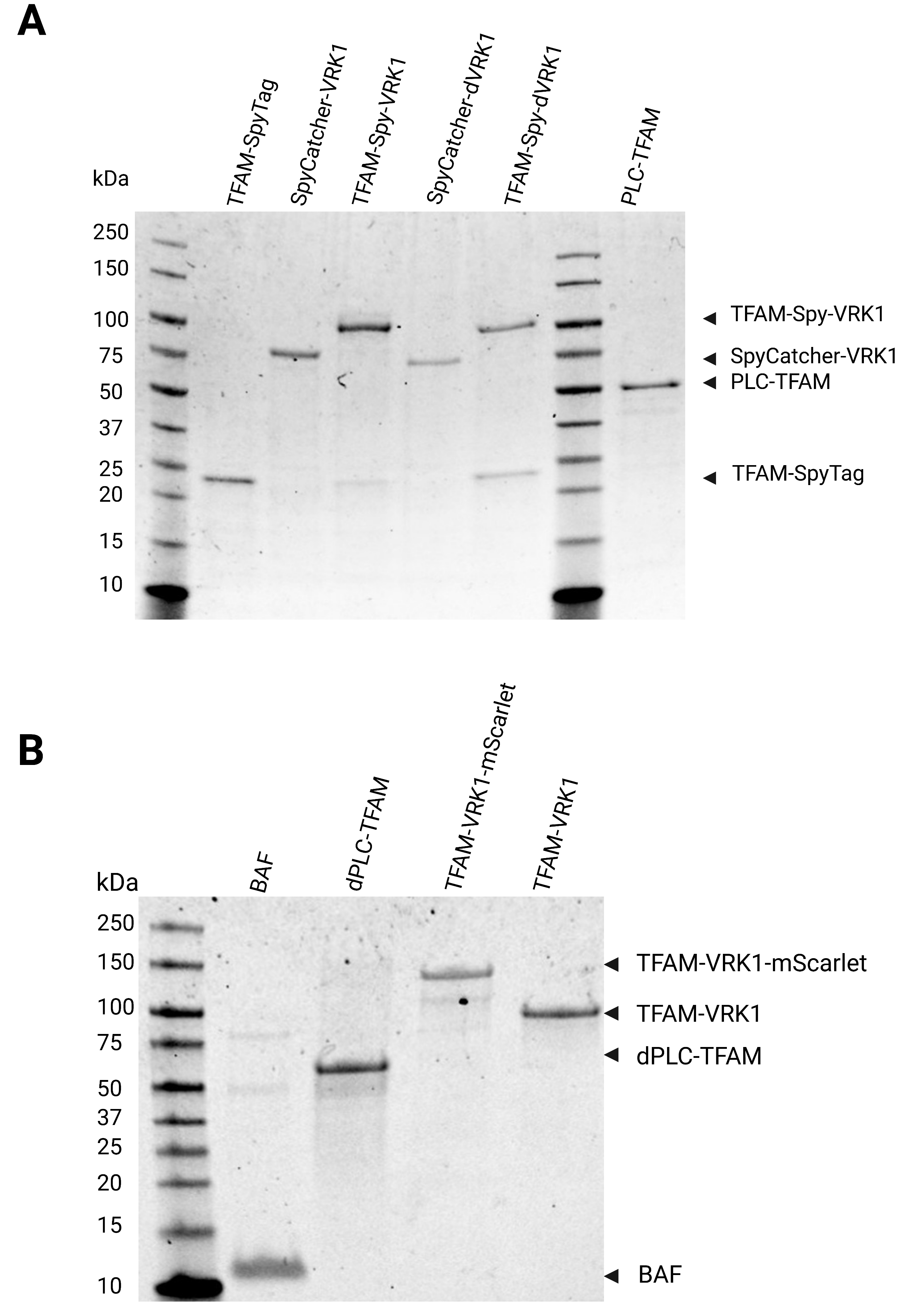
**

**Figure S3**: SDS-PAGE stained with Coomassie Brilliant Blue. **A**) One μg of SpyCatcher-VRK1 or SpyCatcher-dVRK1 were either loaded separately on the gel or after pre-incubated for 15 min with 1 μg TFAM-SpyTag for covalent bond formation. **B**) One μg of BAF, TFAM-VRK1-mScarlet, TFAM-VRK1, dPLC-TFAM were loaded per lane. The arrowheads on the right indicate the expected height of the protein species.

## *In vitro* TFAM-Spy-VRK1 kinase activity assay gel mobility shift


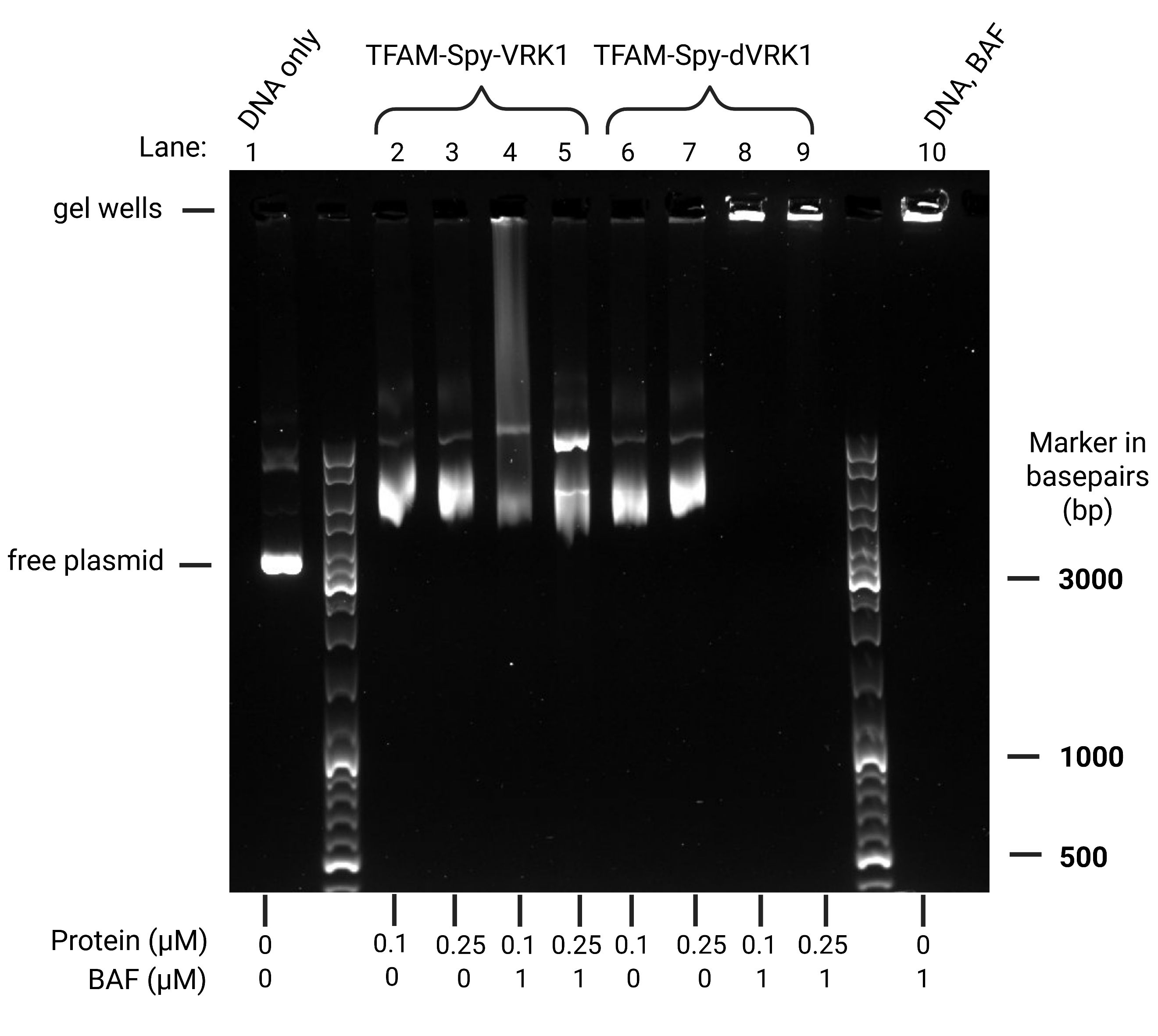


**Figure S4**: Gel mobility shift assay to determine the *in vitro* SpyCatcher-VRK1 kinase activity. TFAM-Spy-VRK1 and TFAM-Spy-dVRK1 were tested at different concentrations (0, 0.1, 0.25 µM per coupling partner). Lanes 1-3, 6, 7 without BAF. Lanes 4, 5, 8, 9, 10 with 1 µM BAF. Marker: GeneRuler DNA Ladder Mix. 50 ng pDNA per well.

## Particle size measurement via DLS


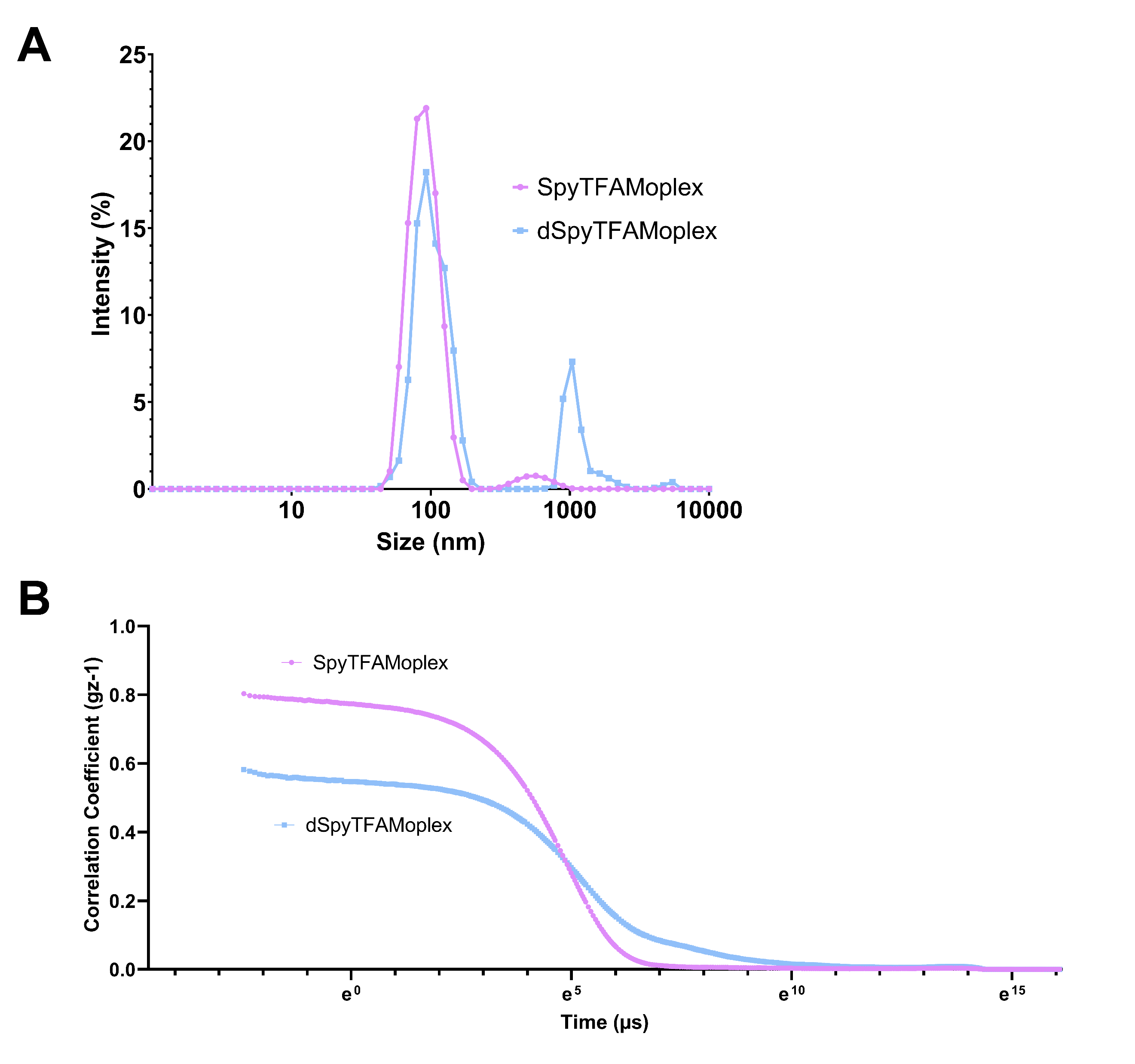


**Figure S5:** Intensity plot (**A**) and Correlation curve (**B**) of DLS measurements of SpyTFAMoplex and dSpyTFAMoplex prepared with 0.6 μM TFAM-SpyTag and 0.4 µM SpyCatcher-VRK1 or 0.4 µM SpyCatcher-dVRK1 with 10 ng mScarlet-pDNA/μL PBS. Respecitvely, each line represents the mean of 3 independent measurements.

## Cytotoxicity assay


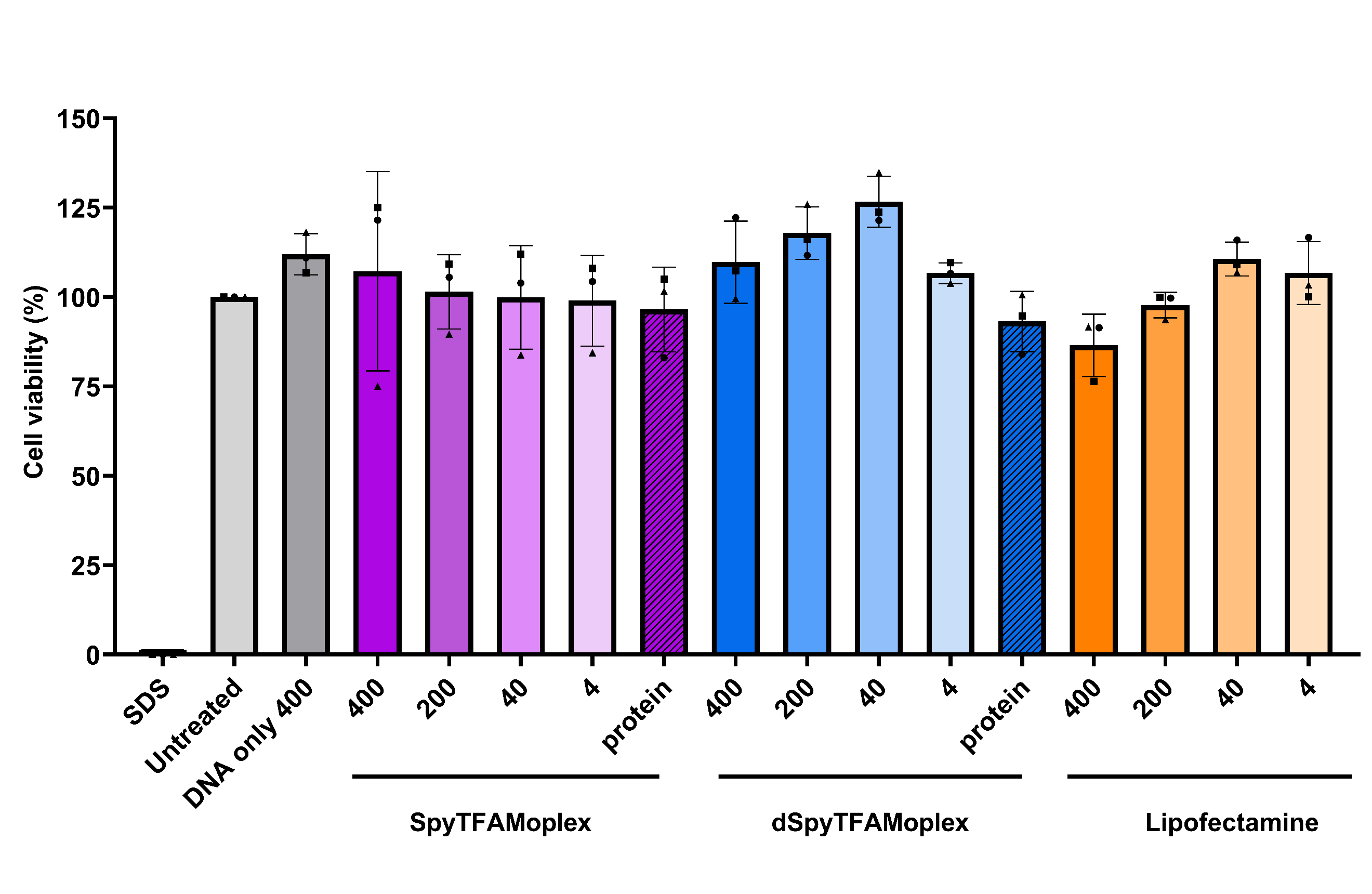


**Figure S6**: Cell viability assay. HeLa cells were incubated with either DNA, proteins, or their combination as TFAMoplexes for one day. Cell viability was assessed with CellTiter 96^®^ AQueous One Solution Cell Proliferation assay (MTS). DNA concentration in ng/mL. Controls: SDS 2% (w/v), untreated, DNA only, proteins only (same concentration as in the 400 ng pDNA/mL condition). SDS 2% was used as a positive toxic control and subtracted from each replicate data. Data was normalized to untreated control. Each symbol shape represents the mean of an independent triplicate experiment. Mean ± SD (N=3). Repeated Measures one-way ANOVA with Dunnett correction did not reveal significant differences in between untreated cells and control and samples.

## EGFP-BAF cells 3 h after transfection with Cy3-DNA at different concentrations


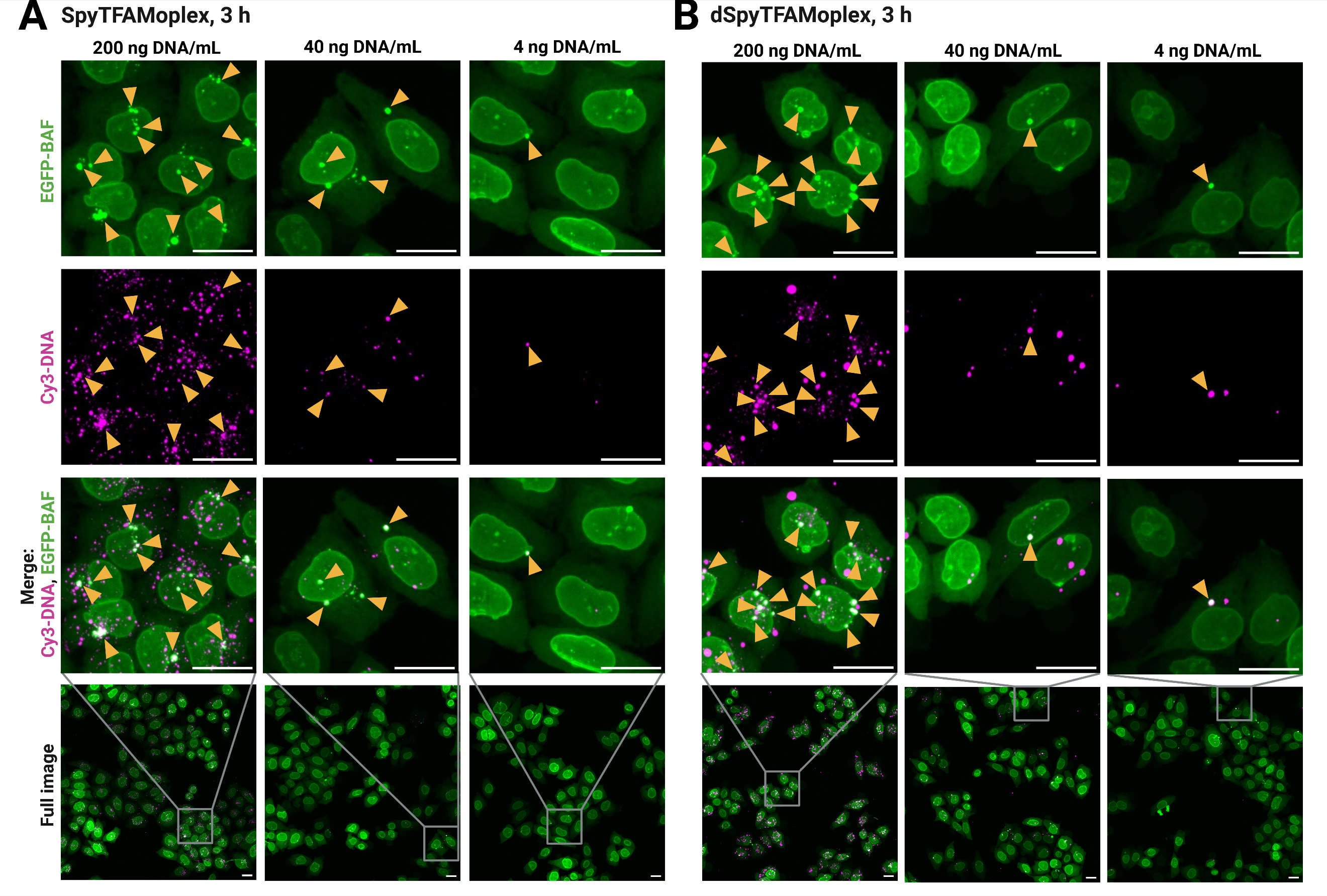


**Figure S7**: EGFP-BAF cells transfected with Cy3-DNA fixed after 3 h. Confocal microscopy zoom-ins and full images displayed as z-projections of maximum intensity of 31 slices with 0.5 µm slice thickness. EGFP-BAF cells were incubated for 30 min with SpyTFAMoplex or dSpyTFAMoplex at different concentrations (200, 40, and 4 ng Cy3-DNA/mL medium). Cells were washed and further incubated until fixation after total 3 h after transfection. Particles were prepared with PLC-TFAM, TFAM-SpyTag and SpyCatcher-VRK1 (**A**) or SpyCatcher-dVRK1 (**B**) and Cy3-DNA. Magenta: Cy3-DNA (intensity: 150 - 600). Green: EGFP-BAF (intensity:150 - 2000). Excitation at 20% laser intensity for 200 ms in all channels. Orange arrowheads indicate colocalization of Cy3 DNA with bright EGFP-BAF foci. Scale bars: 20 µm.

## EGFP-BAF cells 24 h after transfection with Cy3-DNA at different concentrations


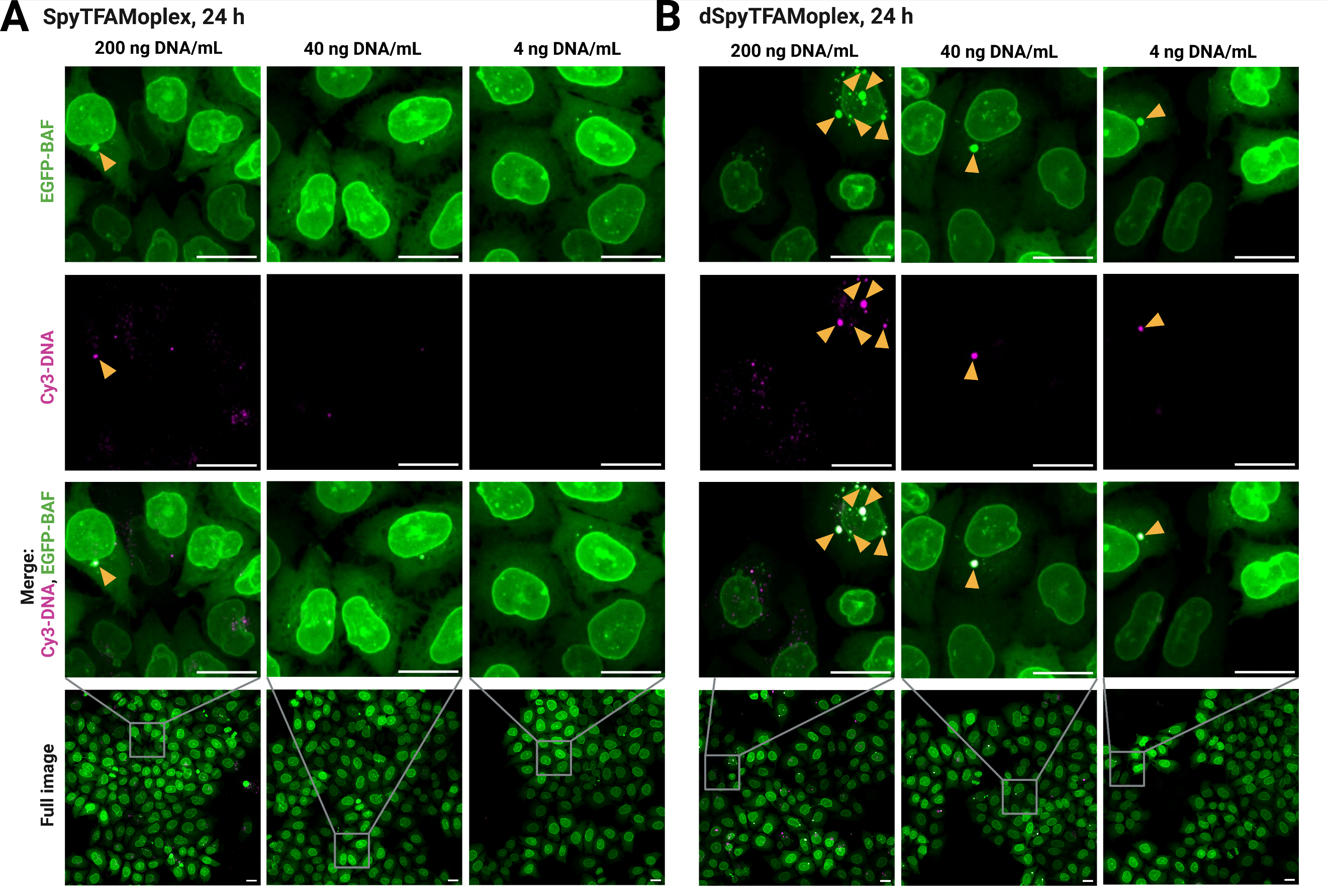


**Figure S8**: EGFP-BAF cells transfected with Cy3-DNA fixed after 24 h. Confocal microscopy crops and full images displayed as z-projections of maximum intensity of 31 slices with 0.5 µm slice thickness. EGFP-BAF cells were incubated for 30 min with SpyTFAMoplex or dSpyTFAMoplex at different concentrations (200, 40, and 4 ng Cy3-DNA/mL medium). Cells were washed and further incubated until fixation after total 24 h post transfection. Particles were prepared with PLC-TFAM, TFAM-SpyTag and SpyCatcher-VRK1 (**A**) or SpyCatcher-dVRK1 (**B**) and Cy3-DNA. Magenta: Cy3-DNA (intensity: 150 - 600). Green: EGFP-BAF (intensity:150 - 2000). Excitation at 20% laser intensity for 200 ms in all channels. Orange arrowheads indicate colocalization of Cy3 DNA with bright EGFP-BAF foci. Scale bars: 20 µm.

## Volcano plots phosphorylation enrichment and protein abundance


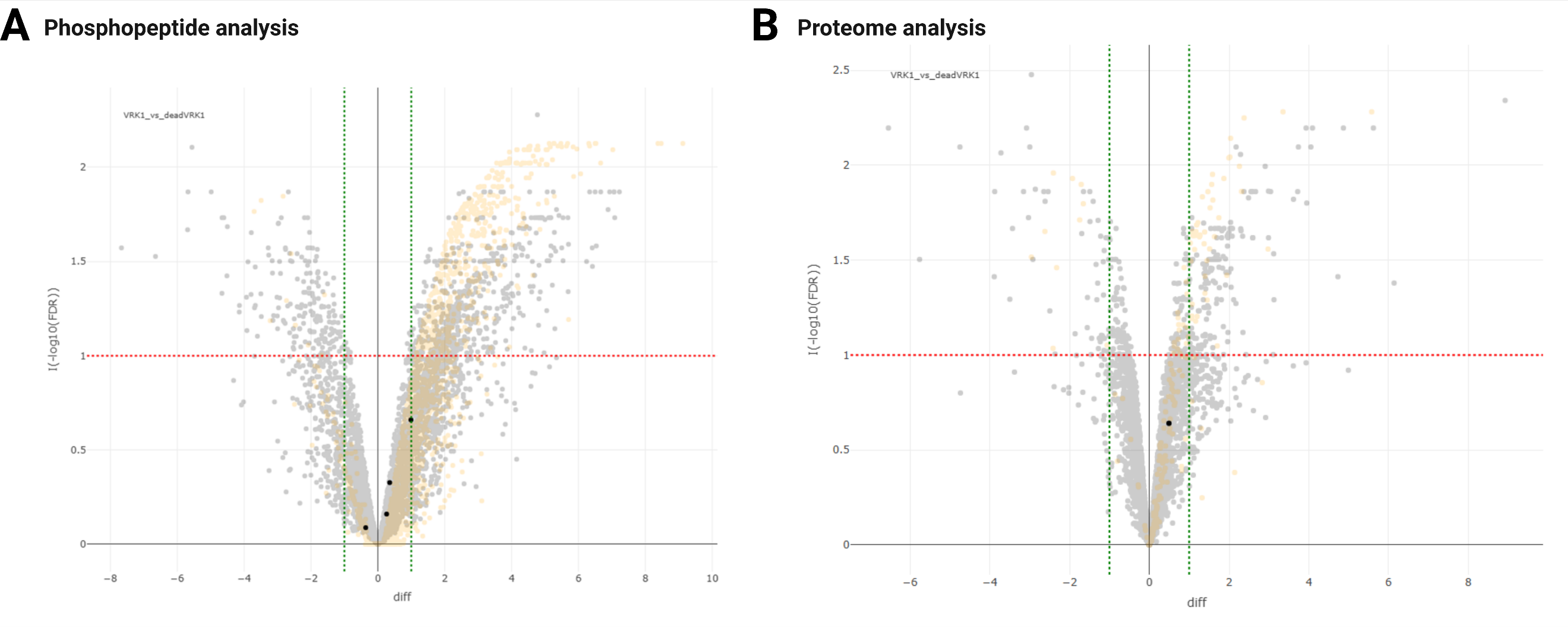


**Figure S9**: Volcano plot showing differential expression analysis of phosphorylation enrichement (**A**) and proteome (**B**) of SpyTFAMoplex (VRK1) treated cells in relation to dSpyTFAMoplex (deadVRK1) treated cells (control). Horizontal axis: difference (contrast), vertical axis: False Discovery Rate (FDR) shown as function of the -log10 (FDR) of the different groups. The red line indicates the -log10(0.1), while the green lines represent the difference of minus and plus 1. Orange dots indicate differences and FDRs estimated using missing value imputation. Highlighted black dots represent the four BAF phosphorylation sites (**A**) or the BAF protein (**B**) (**Table S3**). 1×10^6^ HeLa cells were treated for 4 h with SpyTFAMoplex or dSpyTFAMoplex with 400 ng pDNA/mL medium. The measurement was performed with three independent experiments.

## Anti-emerin AB AF594 background in EGFP-BAF clusters


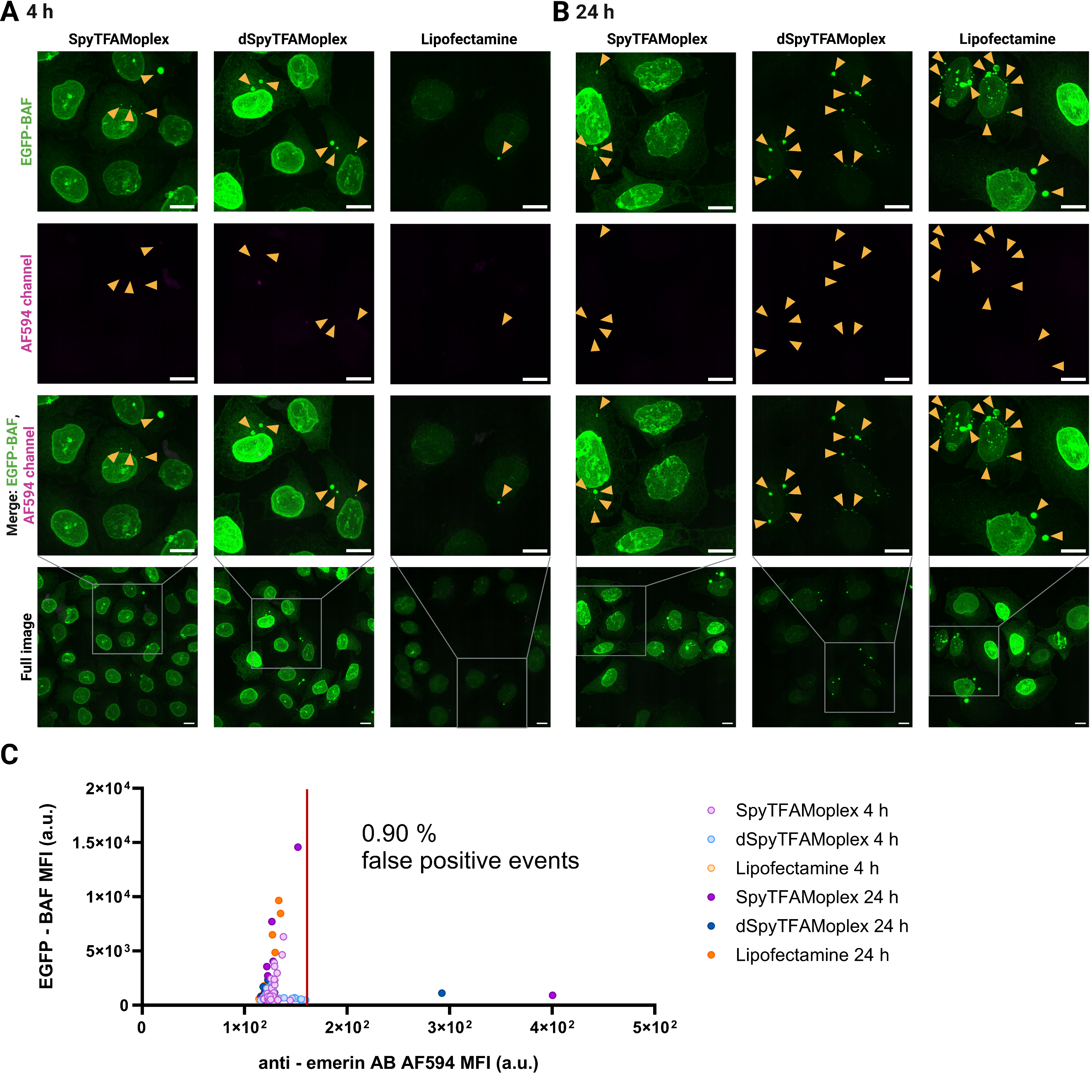


**Figure S10**: AF594 background (no anti-emerin staining applied) in EGFP-BAF HeLa cells transfected with EBFP-pDNA. Confocal microscopy zoom-ins and full images displayed as z-projections of maximum intensity of 31 slices with 0.2 µm slice thickness. Cells were incubated for 30 min with Cy3-DNA in SpyTFAMoplex, dSpyTFAMoplex or Lipofectamine with 400 ng pDNA/mL medium. Cells were washed and further incubated until fixation after 4 h (**A**) and 24 h (**B**). Magenta: AF594 background signal (intensity: 100 - 500). Green: EGFP-BAF (intensity:115 – 500). Scale bars: 10 µm. Orange arrowheads indicate bright EGFP-BAF foci. **C**) EGFP-BAF MFI *vs*. anti-emerin AB AF594 MFI. Each data point represents one EGFP-BAF cluster. The threshold was set at AF594 MFI 160.81. SpyTFAMoplex 4 h: 74 cells, 43 EGFP-BAF clusters; SpyTFAMoplex 24 h: 57 cells, 33 EGFP-BAF clusters; dSpyTFAMoplex 4 h: 41 cells, 56 EGFP-BAF clusters; dSpyTFAMoplex 24 h: 45 cells, 38 EGFP-BAF clusters; Lipofectamine 4 h: 36 cells, 7 EGFP-BAF clusters; Lipofectamine 24 h: 30 cells, 45 EGFP-BAF clusters.

## EGFP-BAF MFI *vs*. anti-emerin AB AF594 MFI in BAF clusters


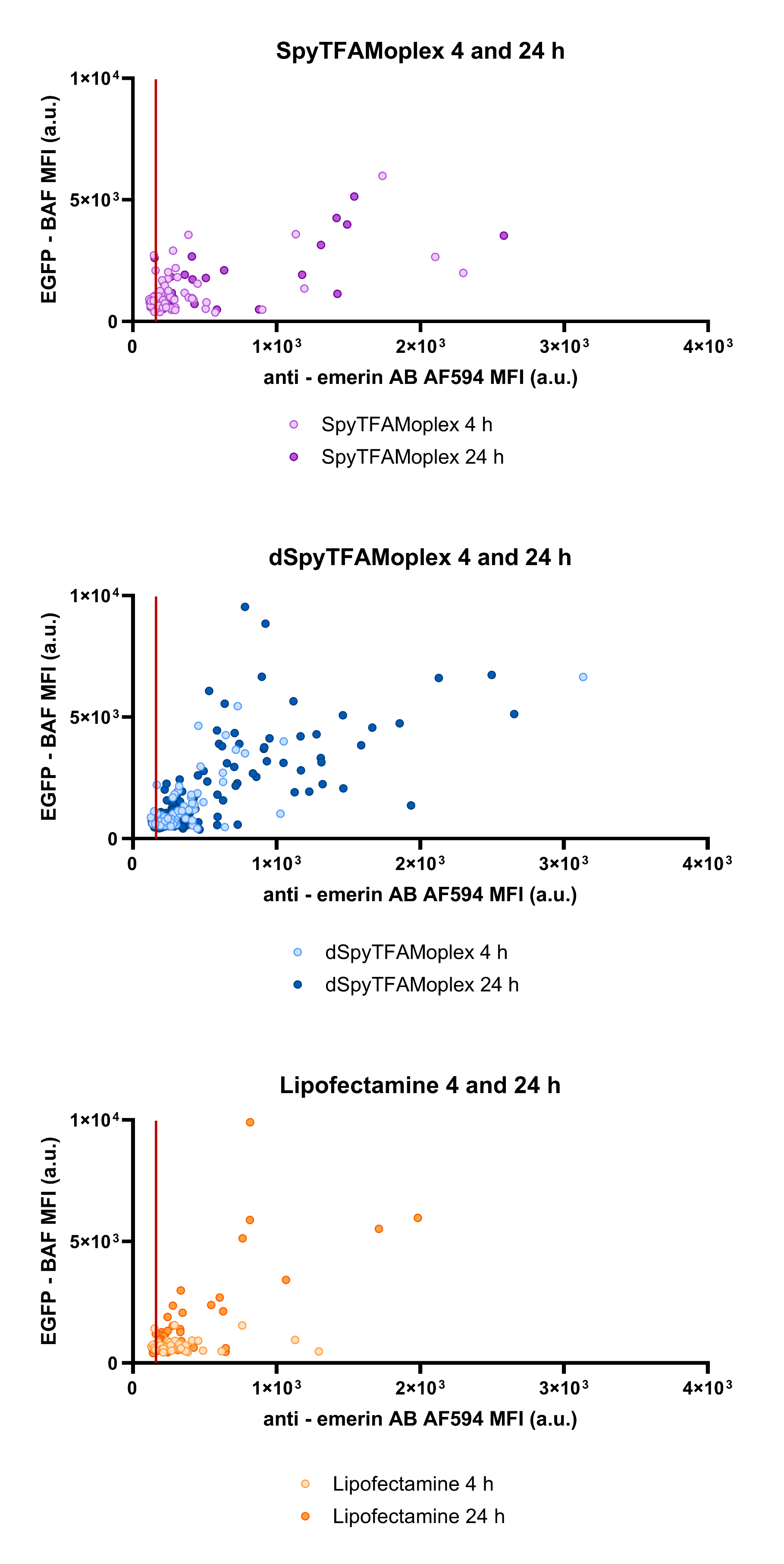


**Figure S11**: Correlation of EGFP-BAF MFI *vs*. anti-emerin AB AF594 MFI. Each data point represents one EGFP-BAF cluster. The threshold was set at AF594 MFI 160.81. SpyTFAMoplex 4 h: 117 cells, 123 EGFP-BAF clusters with average MFI 929 ± 750, SpyTFAMoplex 24 h: 73 cells, 66 EGFP-BAF clusters with average MFI: 1105 ± 990, dSpyTFAMoplex 4 h: 68 cells, 134 EGFP-BAF clusters with average MFI 1012 ± 988, dSpyTFAMoplex 24 h: 65 cells, 375 EGFP-BAF clusters with average MFI 1057 ± 1235, Lipofectamine 4 h: 68 cells, 37 EGFP-BAF clusters with average MFI 747 ± 271, Lipofectamine 24 h: 70 cells, 87 EGFP-BAF clusters with average MFI 1200 ± 1497.

## Lipofectamine and DNA only-treated EGFP-BAF cells counterstained with anti-Cy3 AB


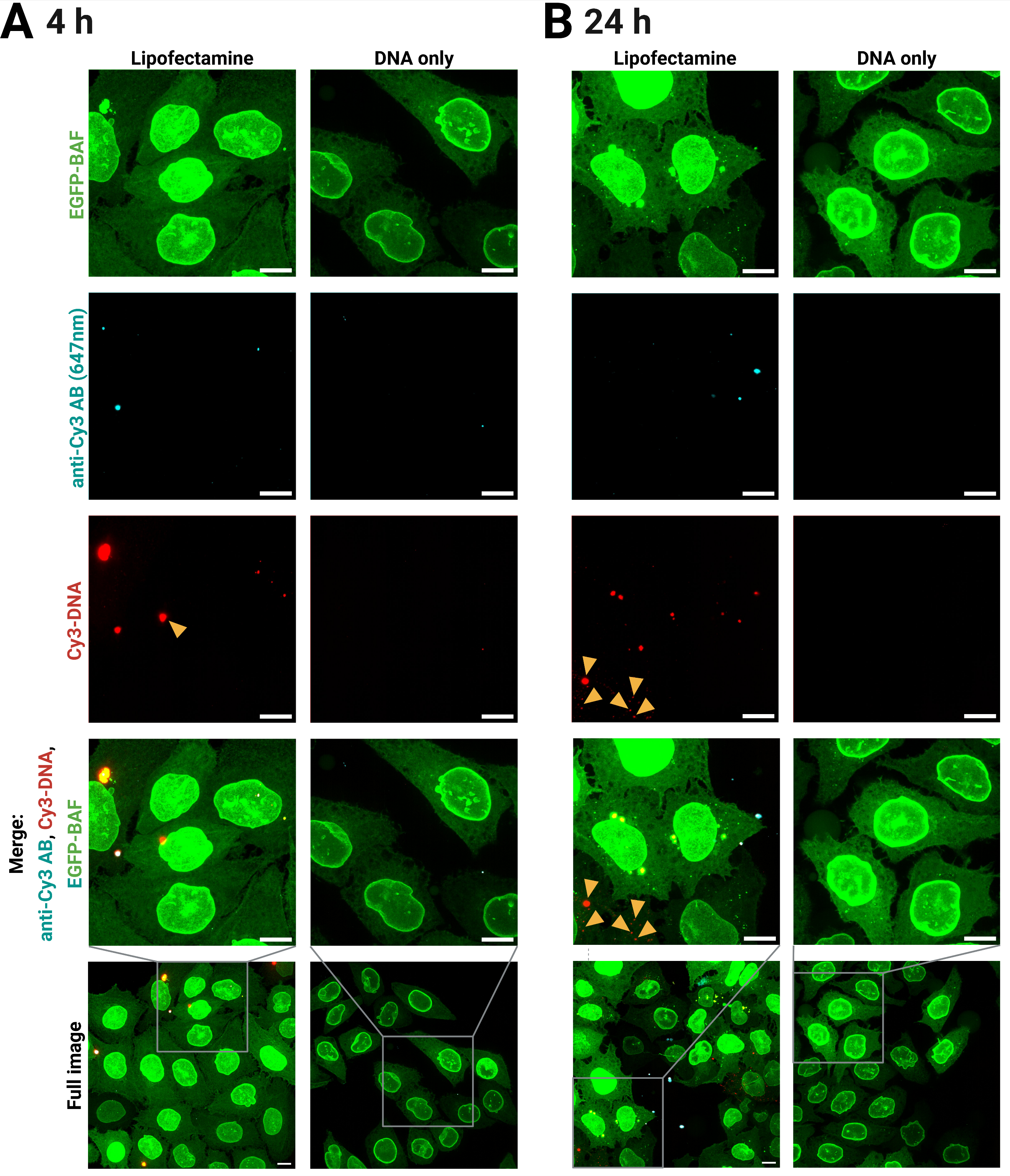


**Figure S12**: Confocal microscopy images of EGFP-BAF cells transfected with Lipofectamine and counterstained with anti-Cy3-AB647. EGFP-BAF cells were transfected with 400 ng Cy3-DNA/mL medium for 30 min, then washed, further incubated and fixed after 4 h (**A**) or 24 h (**B**), and stained. Fluorescence channels displayed as z-projections of maximum intensity of 30 to 38 slices of 0.2 µm slice thickness. Cyan: anti-Cy3-AB (intensity:160 - 500). Red: Cy3-DNA (intensity: 120 - 500). Green: EGFP-BAF (intensity:120 - 500). Scale bars: 10 µm. Orange arrowheads indicate double-negative Cy3-DNA signals.

## Colocalization MFP488-labeled pDNA with Lysotracker DeepRed


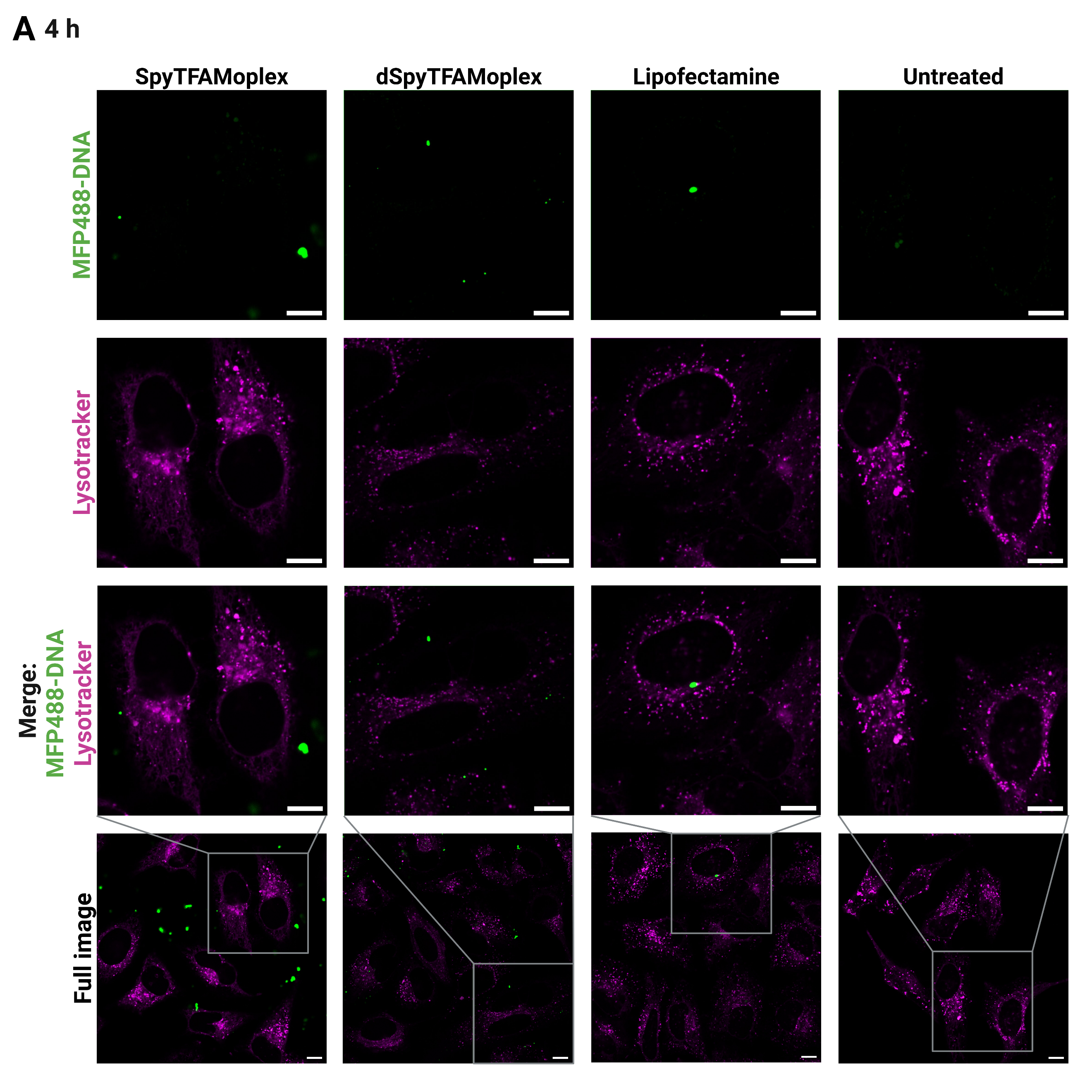


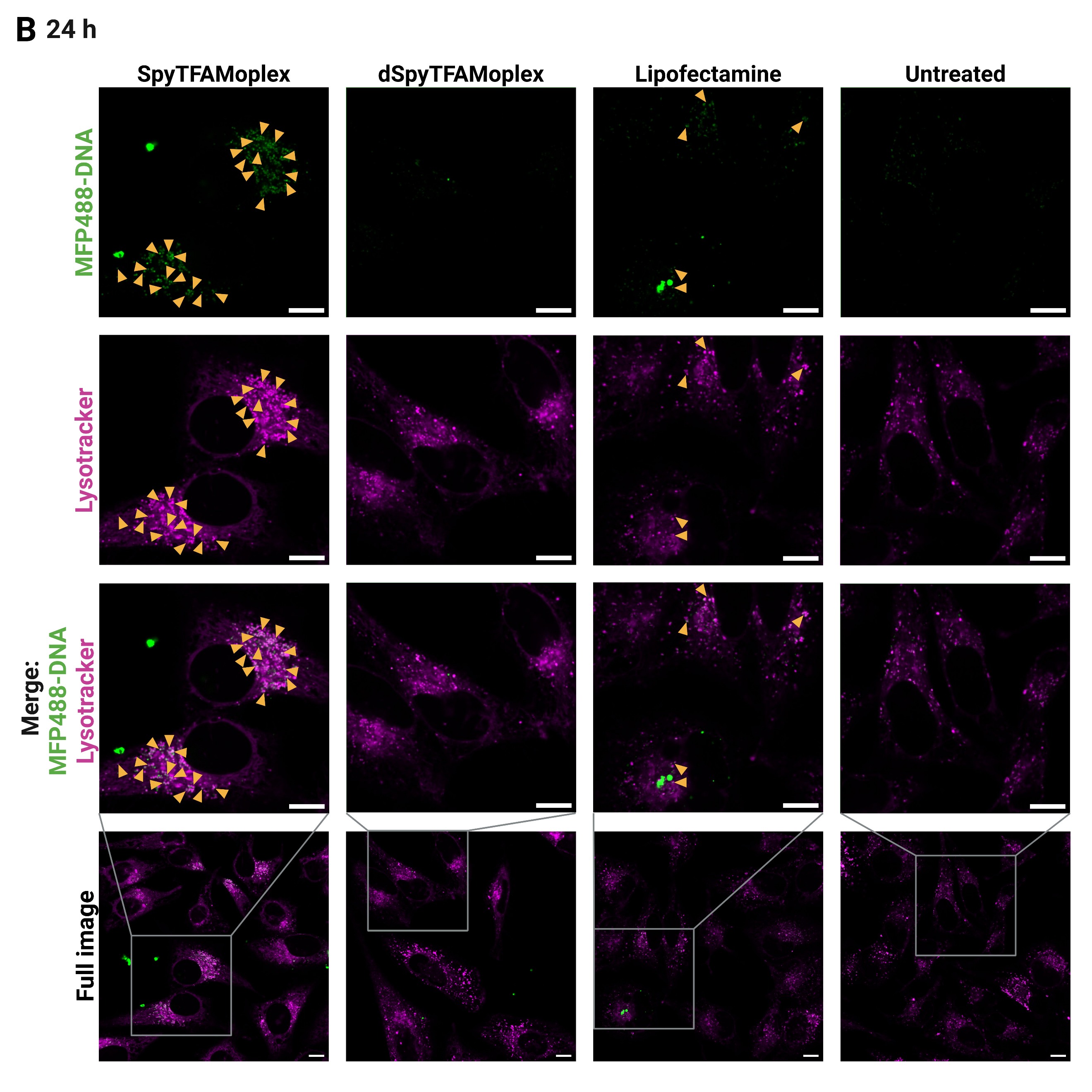


**Figure S13**: HeLa cells transfected with MFP488-labeled EBFP-pDNA and stained with Lysotracker DeepRed. Confocal microscopy full images and zoom-ins of one z-slice. Cells were transfected for 30 min with 400 ng MFP488-labeled pDNA/mL medium, washed and further incubated until imaging after 4 h (**A**) or 24 h (**B**) post transfection. Magenta: Lysotracker DeepRed (intensity: 120-1200). Green: MFP488-pDNA (intensity: 120-800). Orange arrowheads indicate colocalization of MFP488 signal with Lysotracker. Scale bars: 10 µm.

## Tables

**Table S1**: Analysis of live cell imaging of TFAM-VRK1-mScarlet colocalization with EGFP-BAF clusters. Particles were prepared in FBS with PLC-TFAM, TFAM-VRK1-mScarlet and EBFP-pDNA. EGFP-BAF HeLa cells were transfected in FluoroBrite DMEM at 400 ng/mL EBFP-pDNA. Excitation at 5% laser intensity for 100 ms in all channels. Live cell imaging started 30 min after transfection acquiring z-stacks every 5 min. Z-projections of maximum intensities of 18 stacks with 0.5 µm slice thickness of time-resolved images from t=0 min to t=30 min analysed. Intensities: magenta (TFAM-VRK1- mScarlet):120-450, green (EGFP-BAF): 100-300.

| Video | Average  colocalization time* (min) | No. cells | No. representative  events** |
| --- | --- | --- | --- |
| A | 30.45 | 25 | 11 |
| B | 28.89 | 19 | 9 |
| C | 40.00 | 13 | 13 |
| D | 27.14 | 14 | 7 |
| Average or Sum | Average 31.62 ± 5.75 | Total 71 | Total 40 |

*Colocalization time started at onset of BAF clustering until mScarlet signal in EGFP-BAF cluster faded completely back to background. **Representative events comprise EGFP-BAF clustering start before t=20 min.

**Table S2**: ImageJ Macros

| Macro 1 EGFP-BAF cells and clusters  // Set the directory path --> Change to you input and output directory  dir = "….";  output_dir = ";  // Make a list of the files in the input directory list = getFileList(dir);  // Loop through the list of files for (i = 0; i < list.length; i++) {  file = dir + "/" + list[i];    // Check if the file is not a directory and is a nd2 file --> can be changed for any file type  if (File.isDirectory(file) == 0 &&   endsWith(list[i], ".tif")) {    // Open the image and print the files in list to check if the correct files are opened  //run("Bio-Formats Importer", "open=[" + file + "] autoscale display_metadata rois_import=[ROI manager] view=[Hyperstack] stack_order=XYCZT split_channels split_timepoints split_focal_planes");  open(file) ;  print("Opened file: " + list[i]);    // Extract the base name of the input file  baseName = File.nameWithoutExtension;  print(baseName);   rename("MAX" + "/" + baseName);    //ROIs for cells in EGFP_BAF channel  run("Duplicate...", "duplicate channels=2");  rename("cells" + "/" + baseName);  run("Gaussian Blur...", "sigma=4");    //run("Threshold...");  setThreshold(280, 65535, "raw");  setOption("BlackBackground", true);  run("Convert to Mask");  run("Watershed");  rename("mask of cells" + "/" + baseName);  run("Set Measurements...", "area mean standard min centroid add redirect=None decimal=2");  run("Analyze Particles...", "size=200-Infinity circularity=0.40-1.00 display add");    //measure EGFP_BAF signal in ROIS for cells  selectWindow("MAX" + "/" + baseName);  Stack.setChannel(2);  roiManager("Show None");  roiManager("Show All");  run("Clear Results");  roiManager("Measure");   outputFile = output_dir + "/" + baseName + "cells_EGFP_BAF_signal.csv";  saveAs("Results", "outputFile");   outputFile = output_dir + "/" + baseName + "cells.zip";  roiManager("Save", outputFile);    print("ROIS for cells saved: " + outputFile);    selectWindow("mask of cells" + "/" + baseName);  outputFile = output_dir + "/" + baseName + "mask of cells";  saveAs("Tiff", outputFile);    selectWindow("MAX" + "/" + baseName);  outputFile = output_dir + "/" + baseName;  saveAs("Tiff", outputFile);    rename("MAX" + "/" + baseName);    //measure reporter gene expression in ROIS for cells  selectWindow("MAX" + "/" + baseName);  //change to reporter gene expression channel!  Stack.setChannel(1);  roiManager("Show None");  roiManager("Show All");  run("Clear Results");  roiManager("Measure");   outputFile = output_dir + "/" + baseName + "cells_RepGene_express.csv";  saveAs("Results", "outputFile");     rename("MAX" + "/" + baseName);    //close cells roiManager  roiManager("Deselect");  roiManager("Delete");  run("Clear Results");   //segmentation of EGFP_BAF clusters   selectWindow("MAX" + "/" + baseName);  run("Duplicate...", "duplicate channels=2");  rename("EGFP_BAF clusters" + "/" + baseName);    setThreshold(2500, 65535, "raw");  run("Create Mask");  run("Watershed");  run("Set Measurements...", "area mean standard min centroid add redirect=None decimal=2");  run("Analyze Particles...", "size=0.02-200 circularity=0.8-1.00 display add");    //measure EGFP_BAF signal in segemented objects   selectWindow("MAX" + "/" + baseName);  Stack.setChannel(2);  roiManager("Show None");  roiManager("Show All");  run("Clear Results");  roiManager("Measure");   outputFile = output_dir + "/" + baseName + "EGFP_BAF clusters.csv";  saveAs("Results", "outputFile");   outputFile = output_dir + "/" + baseName + "EGFP_BAF clusters.zip";  roiManager("Save", outputFile);    print("ROIs for clusters saved: " + outputFile);    //close clusters roiManager   roiManager("Deselect");  roiManager("Delete");  run("Clear Results");   // Close the images  run("Close All");   // Check if any images are still open and print a message  if (isOpen("Any")) {  print("There are still open images.");  } else {  print("All images closed.");  }   } } |
| --- |
| Macro 2 EGFP-BAF cells  // Set the directory path --> Change to you input and output directory  dir = "….";  output_dir = " ";  // Make a list of the files in the input directory  list = getFileList(dir);  // Loop through the list of files  for (i = 0; i < list.length; i++) {  file = dir + "/" + list[i];  // Check if the file is not a directory and is a nd2 file --> can be changed for any file type  if (File.isDirectory(file) == 0 &&  endsWith(list[i], ".tif")) {    // Open the image and print the files in list to check if the correct files are opened  open(file) ;  print("Opened file: " + list[i]);  // Extract the base name of the input file  baseName = File.nameWithoutExtension;  print(baseName);  run("Duplicate...", "duplicate channels=2");  rename(baseName + "_EGFP");  run("Gaussian Blur...", "sigma=4");  //run("Threshold...");  setThreshold(130, 65535, "raw");  setOption("BlackBackground", true);  run("Convert to Mask");  run("Watershed");  rename(baseName + "_mask_of_cells");  run("Set Measurements...", "area mean standard min centroid add redirect=None decimal=2");  run("Analyze Particles...", "size=200-Infinity circularity=0.40-1.00 display add");  //measure EGFP_BAF signal in ROIS for cells  selectWindow(baseName + ".tif");  Stack.setChannel(2);  roiManager("Show None");  roiManager("Show All");  run("Clear Results");  roiManager("Measure");  outputFile = output_dir + "/" + baseName + "_cells_EGFP_BAF_signal.csv";  saveAs("Results", "outputFile");  outputFile = output_dir + "/" + baseName + "_cells.zip";  roiManager("Save", outputFile);  print("ROIS for cells saved: " + outputFile);  selectWindow(baseName + "_mask_of_cells");  outputFile = output_dir + "/" + baseName + "_mask_of_cells";  saveAs("Tiff", outputFile);  //close clusters roiManager  roiManager("Deselect");  roiManager("Delete");  run("Clear Results");  // Close the images  run("Close All");  // Check if any images are still open and print a message  if (isOpen("Any")) {  print("There are still open images.");  } else {  print("All images closed.");  }  }  } |
| Macro 3 EGFP-BAF clusters with emerin  // Set the directory path --> Change to you input and output directory  dir = "….";  output_dir = ";  // Make a list of the files in the input directory  list = getFileList(dir);  // Loop through the list of files  for (i = 0; i < list.length; i++) {  file = dir + "/" + list[i];  // Check if the file is not a directory and is a nd2 file --> can be changed for any file type  if (File.isDirectory(file) == 0 &&  endsWith(list[i], ".tif")) {    open(file) ;    // Extract the base name of the input file  tifName = File.nameWithoutExtension;  print(tifName);    run("Duplicate...", "duplicate channels=2");  rename("dupEGFP - to process - " + tifName);  run("Gaussian Blur...", "sigma=2");  //run("Threshold...");  setThreshold(350, 65535, "raw");  run("Set Measurements...", "area mean min centroid add redirect=None decimal=2");  run("Analyze Particles...", "size=0.05-100 circularity= 0.95 -1.00 display add");  outputFile = output_dir + "/" + tifName + " EGFP_BAF_objects.zip";  roiManager("Save", outputFile);  run("Clear Results");  //measure EGFP signal in segemented objects  selectWindow(tifName + ".tif");  run("Duplicate...", "duplicate channels=2");  rename("dup_EGFP_" + tifName);  roiManager("Show All");  roiManager("Measure");  outputFile = output_dir + "/" + tifName + "EGFP_BAF.csv";  saveAs("Results", outputFile);  run("Clear Results");  //measure AB signal in segemented objects  selectWindow(tifName + ".tif");  run("Duplicate...", "duplicate channels=1");  rename("dup_emerinAB - " + tifName);    roiManager("Show All");  roiManager("Measure");  outputFile = output_dir + "/" + tifName + "emerinAB.csv";  saveAs("Results", outputFile);  run("Clear Results");  roiManager("Delete");    // Close the images  run("Close All");    }  } |

**Table S3**: BAF phosphorylation status and protein abundance. The measurement was performed with three independent experiments. Difference represents the phosphorylation enrichment or protein abundance, respectively, of SpyTFAMoplex treated cells in relation to dSpyTFAMoplex treated cells (control). Significance was determined with the false discovery rate (FDR) and represented as -log10(FDR) in the volcano plot in **Figure S9**.

| Index | Phospho-rylation site | Contrast* | Difference** | FDR | Difference protein** | FDR  protein | Integrated difference** | Integrated  FDR |
| --- | --- | --- | --- | --- | --- | --- | --- | --- |
| O75531 BAF HUMAN S22 | S22 | VRK1_vs_deadVRK1 | 0.99 | 0.22 | 0.49 | 0.23 | 0.50 | 0.32 |
| O75531 BAF HUMAN S4 | S4 | VRK1_vs_deadVRK1 | -0.36 | 0.82 | 0.49 | 0.23 | -0.86 | 0.80 |
| O75531 BAF HUMAN T2 | T2 | VRK1_vs_deadVRK1 | 0.26 | 0.69 | 0.49 | 0.23 | -0.23 | 0.88 |
| O75531 BAF HUMAN T3 | T3 | VRK1_vs_deadVRK1 | 0.35 | 0.47 | 0.49 | 0.23 | -0.14 | 0.91 |

*Contrast between SpyTFAmoplex (VRK1) and dSpyTFAMoplex (deadVRK1) analysed

**Difference between SpyTFAMoplex and dSpyTFAMoplex condition calculated, *i.e.* enriched phosphorylation or protein abundance. Positive value: enriched for SpyTFAMoplex. Negative value: enriched for dSpyTFAMoplex.

**Table S4**: DNA sequences, molecular weight and molar extinction coefficient (ε) of constructs. The mutant TFAM A105C, V109C was used in the TFAM-VRK1, TFAM-VRK1-mScarlet and TFAM-SpyTag constructs and is specified as TFAM for simplicity.

| Construct | DNA sequence | Mw (Da) | ε  (M^-1^ cm^-1^) | Comments |
| --- | --- | --- | --- | --- |
| MBP-PLC-TFAM | ATGGGTAGCAGCCATCATCATCATCACCACCAAGCTTcgatGAAGATCGAAGAAGGTAAACTGGTAATCTGGATTAACGGCGATAAAGGCTATAACGGTCTCGCTGAAGTCGGTAAGAAATTCGAGAAAGATACCGGAATTAAAGTCACCGTTGAGCATCCGGATAAACTGGAAGAGAAATTCCCACAGGTTGCGGCAACTGGCGATGGCCCTGACATTATCTTCTGGGCACACGACCGCTTTGGTGGCTACGCTCAATCTGGCCTGTTGGCTGAAATCACCCCGGACAAAGCGTTCCAGGACAAGCTGTATCCGTTTACCTGGGATGCCGTACGTTACAACGGCAAGCTGATTGCTTACCCGATCGCTGTTGAAGCGTTATCGCTGATTTATAACAAAGATCTGCTGCCGAACCCGCCAAAAACCTGGGAAGAGATCCCGGCGCTGGATAAAGAACTGAAAGCGAAAGGTAAGAGCGCGCTGATGTTCAACCTGCAAGAACCGTACTTCACCTGGCCGCTGATTGCTGCTGACGGGGGTTATGCGTTCAAGTATGAAAACGGCAAGTACGACATTAAAGACGTGGGCGTGGATAACGCTGGCGCGAAAGCGGGTCTGACCTTCCTGGTTGACCTGATTAAAAACAAACACATGAATGCAGACACCGATTACTCCATCGCAGAAGCTGCCTTTAATAAAGGCGAAACAGCGATGACCATCAACGGCCCGTGGGCATGGTCCAACATCGACACCAGCAAAGTGAATTATGGTGTAACGGTACTGCCGACCTTCAAGGGTCAACCATCCAAACCGTTCGTTGGCGTGCTGAGCGCAGGTATTAACGCCGCCAGTCCGAACAAAGAGCTGGCAAAAGAGTTCCTCGAAAACTATCTGCTGACTGATGAAGGTCTGGAAGCGGTTAATAAAGACAAACCGCTGGGTGCCGTAGCGCTGAAGTCTTACGAGGAAGAGTTGGCGAAAGATCCACGTATTGCCGCCACCATGGAAAACGCCCAGAAAGGTGAAATCATGCCGAACATCCCGCAGATGTCCGCTTTCTGGTATGCCGTGCGTACTGCGGTGATCAACGCCGCCAGCGGTCGTCAGACTGTCGATGAAGCCCTGAAAGACGCGCAGACTAATTCGAGCTCGAACAACAACAACAATAACAATAACAACAACCTCGGGctgcaGgCTTCAAGCGAAAATCTGTATTTCCAATGGAGCGCGGACAATCCGACAAACACTGATGTAAACACCCATTATTGGCTTTTCAAGCAGGCTGAGAAAATTTTAGCAAAAGACGTGAATCATATGCGCGCAAACCTGATGAACGAACTTAAGAAGTTTGATAAACAAATAGCCCAGGGAATTTACGATGCCGACCACAAAAACCCCTATTATGACACTAGCACATTTCTGTCCCACTTCTATAACCCAGATCGCGATAACACGTACTTGCCTGGATTCGCAAATGCAAAGATTACGGGCGCTAAATATTTCAATCAAAGTGTTACGGACTATCGAGAAGGTAAATTCGATACAGCCTTCTACAAATTAGGTCTGGCCATCCACTATTACACAGATATTAGTCAACCTATGCACGCGAATAACTTCACGGCAATTAGCTATCCTCCGGGATATCATTGTGCATACGAAAACTACGTTGATACCATTAAACACAACTACCAGGCTACAGAAGATATGGTTGCCAAACGGTTTTGCAGCGACGACGTTAAAGATTGGTTATATGAAAACGCCAAACGTGCTAAAGCAGATTATCCGAAGATAGTAAACGCGAAAACAAAAAAAAGCTACCTCGTGGGAAACTCTGAATGGAAAAAGGATACGGTCGAACCTACAGGAGCTCGCTTACGTGATTCACAGCAGACGTTAGCCGGGTTCTTAGAATTTTGGTCTAAAAAGACAAATGAAAGCGGTGGTTCTGGTGGATCGGGATCCatgtcatctgtcttgGCAAGTTGTCCAAAGAAACCTGTAAGTTCTTACCTTCGATTTTCTAAAGAACAACTACCCATATTTAAAGCTCAGAACCCAGATGCAAAAACTACAGAACTAATTAGAAGAATTGCCCAGCGTTGGAGGGAACTTCCTGATTCAAAGAAAAAAATATATCAAGATGCTTATAGGGCGGAGTGGCAGGTATATAAAGAAGAGATAAGCAGATTTAAAGAACAGCTAACTCCAAGTCAGATTATGTCTTTGGAAAAAGAAATCATGGACAAACATTTAAAAAGGAAAGCTATGACAAAAAAAAAAGAGTTAACACTGCTTGGAAAACCAAAAAGACCTCGTTCAGCTTATAACGTTTATGTAGCTGAAAGATTCCAAGAAGCTAAGGGTGATTCACCGCAGGAAAAGCTGAAGACTGTAAAGGAAAACTGGAAAAATCTGTCTGACTCTGAAAAGGAATTATATATTCAGCATGCTAAAGAGGACGAAACTCGTTATCATAATGAAATGAAGTCTTGGGAAGAACAAATGATTGAAGTTGGACGAAAGGATCTTCTACGTCGCACAATAAAGAAACAACGAAAATATGGTGCTGAGGAGTGTTaa | 97,618 | 159,060 | Produced as previously described [1] |
| PLC-TFAM | TGGAGCGCGGACAATCCGACAAACACTGATGTAAACACCCATTATTGGCTTTTCAAGCAGGCTGAGAAAATTTTAGCAAAAGACGTGAATCATATGCGCGCAAACCTGATGAACGAACTTAAGAAGTTTGATAAACAAATAGCCCAGGGAATTTACGATGCCGACCACAAAAACCCCTATTATGACACTAGCACATTTCTGTCCCACTTCTATAACCCAGATCGCGATAACACGTACTTGCCTGGATTCGCAAATGCAAAGATTACGGGCGCTAAATATTTCAATCAAAGTGTTACGGACTATCGAGAAGGTAAATTCGATACAGCCTTCTACAAATTAGGTCTGGCCATCCACTATTACACAGATATTAGTCAACCTATGCACGCGAATAACTTCACGGCAATTAGCTATCCTCCGGGATATCATTGTGCATACGAAAACTACGTTGATACCATTAAACACAACTACCAGGCTACAGAAGATATGGTTGCCAAACGGTTTTGCAGCGACGACGTTAAAGATTGGTTATATGAAAACGCCAAACGTGCTAAAGCAGATTATCCGAAGATAGTAAACGCGAAAACAAAAAAAAGCTACCTCGTGGGAAACTCTGAATGGAAAAAGGATACGGTCGAACCTACAGGAGCTCGCTTACGTGATTCACAGCAGACGTTAGCCGGGTTCTTAGAATTTTGGTCTAAAAAGACAAATGAAAGCGGTGGTTCTGGTGGATCGGGATCCatgtcatctgtcttgGCAAGTTGTCCAAAGAAACCTGTAAGTTCTTACCTTCGATTTTCTAAAGAACAACTACCCATATTTAAAGCTCAGAACCCAGATGCAAAAACTACAGAACTAATTAGAAGAATTGCCCAGCGTTGGAGGGAACTTCCTGATTCAAAGAAAAAAATATATCAAGATGCTTATAGGGCGGAGTGGCAGGTATATAAAGAAGAGATAAGCAGATTTAAAGAACAGCTAACTCCAAGTCAGATTATGTCTTTGGAAAAAGAAATCATGGACAAACATTTAAAAAGGAAAGCTATGACAAAAAAAAAAGAGTTAACACTGCTTGGAAAACCAAAAAGACCTCGTTCAGCTTATAACGTTTATGTAGCTGAAAGATTCCAAGAAGCTAAGGGTGATTCACCGCAGGAAAAGCTGAAGACTGTAAAGGAAAACTGGAAAAATCTGTCTGACTCTGAAAAGGAATTATATATTCAGCATGCTAAAGAGGACGAAACTCGTTATCATAATGAAATGAAGTCTTGGGAAGAACAAATGATTGAAGTTGGACGAAAGGATCTTCTACGTCGCACAATAAAGAAACAACGAAAATATGGTGCTGAGGAGTGTTaa | 52,857 | 91,220 | Sequence corresponding to active PLC-TFAM (MBP cleaved off by TEV protease)  Produced as previously described [1] |
| dPLC-TFAM | **g**GGAGCGCGGACAATCCGACAAACACTGATGTAAACACCCATTATTGGCTTTTCAAGCAGGCTGAGAAAATTTTAGCAAAAGACGTGAATCATATGCGCGCAAACCTGATGAACGAACTTAAGAAGTTTGATAAACAAATAGCCCAGGGAATTTACGATGCCGACCACAAAAACCCCTATTATGACACTAGCACATTTCTGTCCCACTTCTATAACCCAGATCGCGATAACACGTACTTGCCTGGATTCGCAAATGCAAAGATTACGGGCGCTAAATATTTCAATCAAAGTGTTACGGACTATCGAGAAGGTAAATTCGATACAGCCTTCTACAAATTAGGTCTGGCCATCCACTATTACACAGATATTAGTCAACCTATGCACGCGAATAACTTCACGGCAATTAGCTATCCTCCGGGATATCATTGTGCATACGAAAACTACGTTGATACCATTAAACACAACTACCAGGCTACAGAAGATATGGTTGCCAAACGGTTTTGCAGCGACGACGTTAAAGATTGGTTATATGAAAACGCCAAACGTGCTAAAGCAGATTATCCGAAGATAGTAAACGCGAAAACAAAAAAAAGCTACCTCGTGGGAAACTCTGAATGGAAAAAGGATACGGTCGAACCTACAGGAGCTCGCTTACGTGATTCACAGCAGACGTTAGCCGGGTTCTTAGAATTTTGGTCTAAAAAGACAAATGAAAGCGGTGGTTCTGGTGGATCGGGATCCatgtcatctgtcttgGCAAGTTGTCCAAAGAAACCTGTAAGTTCTTACCTTCGATTTTCTAAAGAACAACTACCCATATTTAAAGCTCAGAACCCAGATGCAAAAACTACAGAACTAATTAGAAGAATTGCCCAGCGTTGGAGGGAACTTCCTGATTCAAAGAAAAAAATATATCAAGATGCTTATAGGGCGGAGTGGCAGGTATATAAAGAAGAGATAAGCAGATTTAAAGAACAGCTAACTCCAAGTCAGATTATGTCTTTGGAAAAAGAAATCATGGACAAACATTTAAAAAGGAAAGCTATGACAAAAAAAAAAGAGTTAACACTGCTTGGAAAACCAAAAAGACCTCGTTCAGCTTATAACGTTTATGTAGCTGAAAGATTCCAAGAAGCTAAGGGTGATTCACCGCAGGAAAAGCTGAAGACTGTAAAGGAAAACTGGAAAAATCTGTCTGACTCTGAAAAGGAATTATATATTCAGCATGCTAAAGAGGACGAAACTCGTTATCATAATGAAATGAAGTCTTGGGAAGAACAAATGATTGAAGTTGGACGAAAGGATCTTCTACGTCGCACAATAAAGAAACAACGAAAATATGGTGCTGAGGAGTGTTaa | 52,728 | 85,720 | The point mutation W52G in the active site of PLC is marked in bold  Produced as previously described [1] |
| TFAM-VRK1 | ATGGGGAGtTCACACCATCATCACCACCAcGGATCTGGTAGTATGAGTTCAGTGCTGGCTAGCTGTCCGAAAAAACCAGTCTCTTCATATCTGCGTTTTTCAAAAGAGCAGTTGCCAATCTTTAAGGCCCAAAATCCAGATGCGAAAACAACTGAGCTGATTAGACGCATAGCGCAACGGTGGAGAGAACTGCCGGACTCCAAGAAGAAGATTTATCAGGACGCGTATCGCTGTGAGTGGCAATGCTATAAAGAAGAAATATCGCGTTTCAAAGAACAGCTGACCCCTAGTCAGATTATGTCCCTTGAGAAAGAAATCATGGATAAACACCTGAAACGAAAAGCAATGACCAAGAAAAAAGAATTAACCTTACTGGGAAAACCAAAGCGGCCGCGCAGTGCATACAATGTTTATGTGGCTGAACGGTTTCAAGAGGCAAAAGGCGATTCTCCTCAGGAGAAACTGAAAACGGTTAAAGAAAATTGGAAGAACCTCTCCGATTCAGAGAAGGAACTGTATATCCAGCACGCTAAAGAGGATGAAACAAGATATCATAACGAAATGAAATCCTGGGAGGAGCAGATGATTGAGGTAGGTCGGAAAGAcCTTCTACGTCGCACTATTAAAAAACAGCGCAAATACGGTGCTGAAGAATGCAGTGGGGGTAGCTCCGGCCGTGGATCCATGCCCCGTGTGAAGGCGGCGCAGGCTGGACGGCAGTCTTCAGCGAAGcgtCACCTCGCGGAGCAGTTTGCAGTTGGAGAAATTATCACTGATATGGCTAAAAAGGAGTGGAAAGTGGGACTGCCTATTGGACAAGGTGGATTTGGTTGTATCTATCTGGCCGACATGAACTCTTCGGAATCTGTGGGCTCAGATGCTCCCTGTGTAGTCAAAGTAGAACCTTCAGATAATGGGCCGCTGTTTACTGAACTGAAATTTTATCAAAGGGCTGCTAAACCTGAACAGATACAAAAATGGATACGGACTCGGAAATTGAAATATCTCGGCGTACCAAAATATTGGGGTAGCGGACTTCATGATAAAAATGGGAAATCGTATCGTTTTATGATAATGGACCGGTTCGGCTCGGACTTACAAAAAATTTACGAGGCGAACGCCAAACGGTTTAGCCGCAAGACTGTATTACAGCTGAGCTTGCGCATTCTgGATATTCTTGAgTATATcCACGAACATGAATATGTTCATGGTGATATTAAGGCAagcAATTTATTATTGAACTATAAGAACCCGGATCAGGTATATTTGGTGGATTACGGTCTGGCATACCGCTACTGCCCGGAGGGAGTACACAAAGAGTATAAGGAAGACCCAAAACGGTGTCATGAcGGAACCATCGAATTTACCTCGATAGACGCACATAACGGTGTCGCGCCCTCACGTCGTGGAGAcCTGGAAATCCTGGGATATTGCATGATTCAGTGGCTGACGGGACACCTTCCgTGGGAGGATAATCTGAAAGATCCTAAGTATGTGCGAGACAGTAAGATTAGATACAGGGAAAATATAGCCAGCCTGATGGATAAATGCTTTCCaGAAAAGAACAAACCGGGAGAAATCGCTAAATATATGGAGACTGTCAAACTTTTGGATTACACCGAGAAACCGCTGTATGAAAACCTCCGCGATATtTTACTACAGGGCCTGAAAGCCATTGGCAGTAAAGATGATGGCAAGTTAGAcCTGTCAGTGGTTGAAAACGGGGGTCTTAAAGCAAAGACAATTACGAAAAAACGAAAGAAAGAGATTGAAGAATCAAAAGAACCAGGCGTTGAAGATACTGAATGGAGCAATACACAGACAGAAGAGGCTATCCAGACGCGTTCCAGAACCCGCAAACGTGTTCAGAAGAGCGGTCCGAAGAAGAAACGGAAAGTATAA | 73,241 | 98,210 | TFAM A105C, V109C  Produced as previously described [1] |
| TFAM-VRK1-mScarlet | ATGAGTTCAGTGCTGGCTAGCTGTCCGAAAAAACCAGTCTCTTCATATCTGCGTTTTTCAAAAGAGCAGTTGCCAATcTTTAAgGCCCAAAATCCaGATGCGAAAACAACTGAgCTGATTAGACGCATAGCGCAACGGTGGAGAGAACTGCCGGACtccAAGAAgAAGATtTATCAGGACGCgTATCGCTGTGAGTGGCAATGCTATAAAGAAGAAATATCGCGTTTCAAAGAACAGCTGACCCCTAGTCAGATTATGTCCCTTGAGAAAGAAATCATGGATAAACACCTGAAACGAAAAGCAATGACCAAGAAAAAAGAATTAACCTTACTGGGAAAACCAAAGCGGCCGCGCAGTGCATACAATGTTTATGTGGCTGAACGGTTTCAAGAGGCAAAAGGCGATTCTCCTCAGGAGAAACTGAAAACGGTTAAAGAAAATTGGAAGAACCTCTCCGATTCAGAGAAGGAACTGTATATCCAGCACGCTAAAGAgGATGAAACAAGATATCATAACGAAATGAAATCCTGGGAGGAGCAGATGATTGAGGTAGGTCGGAAAGAcCTTCTACGTCGCACTATTAAAAAACAGCGCAAATACGGTGCTGAAGAATGCAGTGGGGGTAGCTCCGGCCGTGGATCCATGCCCCGTGTGAAGGCGGCGCAGGCTGGACGGCAGTCTTCAGCGAAGcgtCACCTCGCGGAGCAGTTTGCAGTTGGAGAAATTATCACTGATATGGCTAAAAAGGAGTGGAAAGTGGGACTGCCTATTGGACAAGGTGGATTTGGTTGTATCTATCTGGCCGACATGAACTCTTCGGAATCTGTGGGCTCAGATGCTCCCTGTGTAGTCAAAGTAGAACCTTCAGATAATGGGCCGCTGTTTACTGAACTGAAATTTTATCAAAGGGCTGCTAAACCTGAACAGATACAAAAATGGATACGGACTCGGAAATTGAAATATCTCGGCGTACCAAAATATTGGGGTAGCGGACTTCATGATAAAAATGGGAAATCGTATCGTTTTATGATAATGGACCGGTTCGGCTCGGACTTACAAAAAATTTACGAGGCGAACGCCAAACGGTTTAGCCGCAAGACTGTATTACAGCTGAGCTTGCGCATTCTgGATATTCTTGAgTATATcCACGAACATGAATATGTTCATGGTGATATTAAGGCAagcAATTTATTATTGAACTATAAGAACCCGGATCAGGTATATTTGGTGGATTACGGTCTGGCATACCGCTACTGCCCGGAGGGAGTACACAAAGAGTATAAGGAAGACCCAAAACGGTGTCATGAcGGAACCATCGAATTTACCTCGATAGACGCACATAACGGTGTCGCGCCCTCACGTCGTGGAGAcCTGGAAATCCTGGGATATTGCATGATTCAGTGGCTGACGGGACACCTTCCgTGGGAGGATAATCTGAAAGATCCTAAGTATGTGCGAGACAGTAAGATTAGATACAGGGAAAATATAGCCAGCCTGATGGATAAATGCTTTCCaGAAAAGAACAAACCGGGAGAAATCGCTAAATATATGGAGACTGTCAAACTTTTGGATTACACCGAGAAACCGCTGTATGAAAACCTCCGCGATATtTTACTACAGGGCCTGAAAGCCATTGGCAGTAAAGATGATGGCAAGTTAGAcCTGTCAGTGGTTGAAAACGGGGGTCTTAAAGCAAAGACAATTACGAAAAAACGAAAGAAAGAGATTGAAGAATCAAAAGAACCAGGCGTTGAAGATACTGAATGGAGCAATACACAGACAGAAGAGGCTATCCAGACGCGTTCCAGAACCCGCAAACGTGTTCAGAAGAGCGGTCCGAAGAAGAAACGGAAAGTTGGTGGTGGTGGAGGGAATTCCgggATGGACTCTACTGAAGCCGTTATCAAAGAATTTATGCGCTTTAAGGTACACATGGAGGGATCTATGAATGGTCATGAgTTCGAGATTGAAGGAGAAGGCGAAGGCCGTCCGTATGAAGGaACCCAGACTGCAAAATTGCGTGTGACGAAGGGTGGCCCTTTGCCATTCTCTTGGGATATTTTAAGCCCGCAGTTTATGTACGGGTCTCGGGCGTTTACTAAACACCCTGCTGACATCCCAGATTACTGGAAGCAATCATTTCCAGAGGGCTTTAAATGGGAACGAGTGATGAATTTCGAAGATGGAGGAGCTGTGTCCGTTGCACAGGATACGTCACTTGAAGATGGAACACTGATTTATAAAGTCAAATTGCGAGGGACTAATTTTCCACCAGATGGTCCAGTGATGCAGAAAAAAACGATGGGTTGGGAAGCCTCGACGGAACGCCTGTATCCGGAGGATGTTGTGCTGAAAGGTGATATTAAAATGGCACTGCGTTTGAAAGATGGCGGTAGATATTTGGCAGATTTTAAGACCACATATAGGGCTAAAAAACCTGTTCAGATGCCTGGAGCATTTAACATCGACAGAAAGTTAGATATCACGAGCCACAATGAAGATTATACAGTTGTGGAACAATATGAACGCTCCGTAGCCCGCCACTCTACTtaa | 99,215 | 133,620 | TFAM A105C, V109C  Produced as previously described [1] |
| TFAM-SpyTag | ATGGGTAGCAGCCATCATCATCATCACCACTCTGCAGGAGGTACCGGACAAGCTTTGATGTCATCTGTCTTGGCAAGTTGTCCAAAGAAACCTGTAAGTTCTTACCTTCGATTTTCTAAAGAACAACTACCCATATTTAAAGCTCAGAACCCAGATGCAAAAACTACAGAACTAATTAGAAGAATTGCCCAGCGTTGGAGGGAACTTCCTGATTCAAAGAAAAAAATATATCAAGATGCTTATAGGtgtGAGTGGCAGtgtTATAAAGAAGAGATAAGCAGATTTAAAGAACAGCTAACTCCAAGTCAGATTATGTCTTTGGAAAAAGAAATCATGGACAAACATTTAAAAAGGAAAGCTATGACAAAAAAAAAAGAGTTAACACTGCTTGGAAAACCAAAAAGACCTCGTTCAGCTTATAACGTTTATGTAGCTGAAAGATTCCAAGAAGCTAAGGGTGATTCACCGCAGGAAAAGCTGAAGACTGTAAAGGAAAACTGGAAAAATCTGTCTGACTCTGAAAAGGAATTATATATTCAGCATGCTAAAGAGGACGAAACTCGTTATCATAATGAAATGAAGTCTTGGGAAGAACAAATGATTGAAGTTGGACGAAAGGATCTTCTACGTCGCACAATAAAGAAACAACGAAAATATGGTGCTGAGGAGTGTGGATCCCGTGGTGTTCCACATATTGTGATGGTCGAtGCGTATAAAAGGTATAAATCCTAA | 23,641 | 38,640 | TFAM A105C, V109C |
| SpyCatcher-VRK1 | ATGGGTAGCAGCCATCATCATCATCACCACTCTGCAGGAGGTACCGGACAAGCTTCTGGATCCGGTTCTGTGACCACACTGAGCGGACTGTCGGGAGAACAGGGGCCAAGTGGAGATATGACCACCGAAGAAGATTCTGCTACGCACATAAAGTTTAGTAAGCGTGACGAGGATGGACGTGAACTCGCAGGCGCCACGATGGAGTTACGTGATAGTAGTGGCAAAACTATCTCAACATGGATTTCTGATGGTCACGTTAAGGATTTCTACTTGTATCCAGGTAAGTATACATTCGTCGAAACGGCAGCTCCTGATGGGTATGAGGTGGCGACTCCTATTGAATTTACCGTGAATGAGGACGGCCAGGTTACCGTGGACGGGGAAGCGACGGAAGGCGATGCCCATACGTCTGCAGGAATGCCCCGTGTGAAGGCGGCGCAGGCTGGACGGCAGTCTTCAGCGAAGCGTCACCTCGCGGAGCAGTTTGCAGTTGGAGAAATTATCACTGATATGGCTAAAAAGGAGTGGAAAGTGGGACTGCCTATTGGACAAGGTGGATTTGGTTGTATCTATCTGGCCGACATGAACTCTTCGGAATCTGTGGGCTCAGATGCTCCCTGTGTAGTCAAAGTAGAACCTTCAGATAATGGGCCGCTGTTTACTGAACTGAAATTTTATCAAAGGGCTGCTAAACCTGAACAGATACAAAAATGGATACGGACTCGGAAATTGAAATATCTCGGCGTACCAAAATATTGGGGTAGCGGACTTCATGATAAAAATGGGAAATCGTATCGTTTTATGATAATGGACCGGTTCGGCTCGGACTTACAAAAAATTTACGAGGCGAACGCCAAACGGTTTAGCCGCAAGACTGTATTACAGCTGAGCTTGCGCATTCTGGATATTCTTGAGTATATCCACGAACATGAATATGTTCATGGTGATATTAAGGCAAGCAATTTATTATTGAACTATAAGAACCCGGATCAGGTATATTTGGTGGATTACGGTCTGGCATACCGCTACTGCCCGGAGGGAGTACACAAAGAGTATAAGGAAGACCCAAAACGGTGTCATGACGGAACCATCGAATTTACCTCGATAGACGCACATAACGGTGTCGCGCCCTCACGTCGTGGAGACCTGGAAATCCTGGGATATTGCATGATTCAGTGGCTGACGGGACACCTTCCGTGGGAGGATAATCTGAAAGATCCTAAGTATGTGCGAGACAGTAAGATTAGATACAGGGAAAATATAGCCAGCCTGATGGATAAATGCTTTCCAGAAAAGAACAAACCGGGAGAAATCGCTAAATATATGGAGACTGTCAAACTTTTGGATTACACCGAGAAACCGCTGTATGAAAACCTCCGCGATATTTTACTACAGGGCCTGAAAGCCATTGGCAGTAAAGATGATGGCAAGTTAGACCTGTCAGTGGTTGAAAACGGGGGTCTTAAAGCAAAGACAATTACGAAAAAACGAAAGAAAGAGATTGAAGAATCAAAAGAACCAGGCGTTGAAGATACTGAATGGAGCAATACACAGACAGAAGAGGCTATCCAGACGCGTTCCAGAACCCGCAAACGTGTTCAGAAGAGCGGTCCGAAGAAGAAACGGAAAGTATAA | 60,999 | 74,635 |  |
| SpyCatcher-dVRK1 | ATGGGTAGCAGCCATCATCATCATCACCACTCTGCAGGAGGTACCGGACAAGCTTCTGGATCCGGTTCTGTGACCACACTGAGCGGACTGTCGGGAGAACAGGGGCCAAGTGGAGATATGACCACCGAAGAAGATTCTGCTACGCACATAAAGTTTAGTAAGCGTGACGAGGATGGACGTGAACTCGCAGGCGCCACGATGGAGTTACGTGATAGTAGTGGCAAAACTATCTCAACATGGATTTCTGATGGTCACGTTAAGGATTTCTACTTGTATCCAGGTAAGTATACATTCGTCGAAACGGCAGCTCCTGATGGGTATGAGGTGGCGACTCCTATTGAATTTACCGTGAATGAGGACGGCCAGGTTACCGTGGACGGGGAAGCGACGGAAGGCGATGCCCATACGTCTGGGAATTCTGGAATGCCCCGTGTGAAGGCGGCGCAGGCTGGACGGCAGTCTTCAGCGAAGCGTCACCTCGCGGAGCAGTTTGCAGTTGGAGAAATTATCACTGATATGGCTAAAAAGGAGTGGAAAGTGGGACTGCCTATTGGACAAGGTGGATTTGGTTGTATCTATCTGGCCGACATGAACTCTTCGGAATCTGTGGGCTCAGATGCTCCCTGTGTAGTCAAAGTAGAACCTTCAGATAATGGGCCGCTGTTTACTGAACTGAAATTTTATCAAAGGGCTGCTAAACCTGAACAGATACAAAAATGGATACGGACTCGGAAATTGAAATATCTCGGCGTACCAAAATATTGGGGTAGCGGACTTCATGATAAAAATGGGAAATCGTATCGTTTTATGATAATGGACCGGTTCGGCTCGGACTTACAAAAAATTTACGAGGCGAACGCCAAACGGTTTAGCCGCAAGACTGTATTACAGCTGAGCTTGCGCATTCTGGATATTCTTGAGTATATCCACGAACATGAATATGTTCATGGT**GCA**ATTAAGGCAAGCAATTTATTATTGAACTATAAGAACCCGGATCAGGTATATTTGGTGGATTACGGTCTGGCATACCGCTACTGCCCGGAGGGAGTACACAAAGAGTATAAGGAAGACCCAAAACGGTGTCATGACGGAACCATCGAATTTACCTCGATAGACGCACATAACGGTGTCGCGCCCTCACGTCGTGGAGACCTGGAAATCCTGGGATATTGCATGATTCAGTGGCTGACGGGACACCTTCCGTGGGAGGATAATCTGAAAGATCCTAAGTATGTGCGAGACAGTAAGATTAGATACAGGGAAAATATAGCCAGCCTGATGGATAAATGCTTTCCAGAAAAGAACAAACCGGGAGAAATCGCTAAATATATGGAGACTGTCAAACTTTTGGATTACACCGAGAAACCGCTGTATGAAAACCTCCGCGATATTTTACTACAGGGCCTGAAAGCCATTGGCAGTAAAGATGATGGCAAGTTAGACCTGTCAGTGGTTGAAAACGGGGGTCTTAAAGCAAAGACAATTACGAAAAAACGAAAGAAAGAGATTGAAGAATCAAAAGAACCAGGCGTTGAAGATACTGAATGGAGCAATACACAGACAGAAGAGGCTATCCAGACGCGTTCCAGAACCCGCAAACGTGTTCAGAAGAGCGGTCCGAAGAAGAAACGGAAAGTATAA | 61,142 | 74635 | The point mutation D177A in the active site of VRK1 is marked in bold |
| BAF | ATGACCACTAGTCAAAAACACCGCGATTTTGTGGCCGAGCCTATGGGAGAGAAGCCGGTGGGGTCACTGGCTGGAATTGGGGAAGTTCTTGGTAAGAAGttaGAGGAGAGAGGATTCGATAAGGCTTACGTTGTGCTAGGCCAATTTCTGGTGTTGAAGAAGGACGAGGACCTTTTCCGTGAATGGCTGAAAGATACATGCGGTGCCAATGCAAAGCAGAGTCGTGATTGCTTTGGTTGCCTCAGAGAATGGTGCGATGCATTCCTTtaa | 10,058 | 12,490 | Provided by Steffen Honrath and produced as described in [2] |

## References

[1] M. Burger, S. Kaelin, J.-C. Leroux, The TFAMoplex—Conversion of the mitochondrial transcription factor A into a DNA transfection agent, Adv. Sci. 9 (2022) 2104987, https://doi.org/10.1002/advs.202104987

[2] M. Burger, C. Schmitt-Koopmann, J.-C. Leroux, DNA unchained: two assays to discover and study inhibitors of the DNA clustering function of barrier-to-autointegration factor, Sci. Rep. 10 (2020) 12301, https://doi.org/10.1038/s41598-020-69246-x
